# Supplementary material for: The nucleoid of rapidly growing Escherichia coli localizes close to the inner membrane and is organized by transcription, translation, and cell geometry
Source: Nat Commun. 2025 Apr 20;16:3732. doi: 10.1038/s41467-025-58723-4 (PMC12009437; doi:10.1038/s41467-025-58723-4)
Supplement: Supplementary file 1 — Supplementary information [file 41467_2025_58723_MOESM1_ESM.pdf]

Supplementary Information

The nucleoid of rapidly growing Escherichia coli localizes close to the inner membrane and is organized by transcription, translation and cell geometry

Christoph Spahn[1,2,3*], Stuart Middlemiss[4], Estibaliz Gómez-de-Mariscal[5,6,7], Ricardo Henriques[5,6,7,8], Helge B. Bode[2,9,10,11,12], Séamus Holden[4,13], Mike Heilemann[1*]

[1] Institute of Physical and Theoretical Chemistry, Goethe-University Frankfurt, Frankfurt, Germany

[2] Department of Natural Products in Organismic Interaction, Max Planck Institute for Terrestrial Microbiology, Marburg, Germany

[3] Rudolf Virchow Center for Integrative and Translational Bioimaging, University of Würzburg, 97080 Würzburg, Germany

[4] Centre for Bacterial Cell Biology, Newcastle University Biosciences Institute, Faculty of Medical Sciences, Newcastle upon Tyne NE24AX, United Kingdom

[5] Optical cell biology group, Instituto Gulbenkian de Ciência, 2780-156 Oeiras, Portugal

[6] Optical cell biology group, Gulbenkian Institute of Molecular Medicine, Oeiras, Portugal

[7] AI-driven Optical Biology, Instituto de Tecnologia Química e Biológica António Xavier, Universidade Nova de Lisboa, Oeiras, Portugal

[8] MRC-Laboratory for Molecular Cell Biology, University College London, London, UK

[9] Molecular Biotechnology, Department of Biosciences, Goethe-University Frankfurt, 60438 Frankfurt, Germany

[10] Center for Synthetic Microbiology (SYNMIKRO), Phillips University Marburg, 35043 Marburg, Germany

[11] Senckenberg Gesellschaft für Naturforschung, 60325 Frankfurt, Germany

[12] Department of Chemistry, Phillips University Marburg, 35043 Marburg, Germany

[13] School of Life Sciences, University of Warwick, Gibbet Hill Campus, Coventry, UK

* Corresponding authors

Christoph Spahn (lead contact): Christoph.spahn@uni-wuerzburg.de

Mike Heilemann: heilemann@chemie.uni-frankfurt.de

Supplementary Notes 1-3

Supplementary Figures 1-29

Supplementary Tables 1-7

Supplementary Notes

Supplementary Note 1: The effect of 2D projection in CLSM and SMLM images

Projection effects are omnipresent in 2D imaging [1], with the only notable exception of TIRF imaging. Fluorescence signal detected within a certain 3D volume is projected onto a 2D plane on the sensor (here the camera chip), providing a distorted view of the target's true localization and spatial distribution. The sphero-cylindrical shape of gram-negative bacteria is hereby particularly problematic, as signal above or below the centre plane is projected into the central part of the 2D cross-section. In SMLM, this effect can be reduced by filtering single-molecule localizations by their width, as PSF width increases with axial distance from the focal plane [2]. The resulting projection depth, however, still comprises several hundreds of nanometres. Practically, this results in nucleoids that appear to span the cell cytosol. While this is might be the case occasionally, most cytosol-crossings of nucleoids during fast growth are likely 2D projection artefacts (see **Figure 1**). In widened cells or bacteria with larger diameters, parts of the membrane-attached nucleoid reside outside of the projection volume. As the projection volume is defined by the optics and analysis parameters in SMLM, a larger cell diameter results in images with less DNA signal being projected into the central region of the 2D cross-section. In RID plots, DNA signal thus appears closer to the cell boundaries, while cells with regular rod-shape provide RID plots with a broader intensity distribution (see **Figures 4** and **6** as examples). Despite its sectioning capabilities, confocal imaging is also highly affected by 2D projection. Assuming an emission wavelength of 600 nm, the use of a 1.4 NA oil objective and a pinhole size of 1 Airy unit, the axial resolution of a confocal image would amount ~ 600 nm. As this projection volume is larger than the respective volume in SMLM, nucleoids appear less membrane-associated in confocal images and the resulting population averages (see **Figure 3**).

Supplementary Note 2: Radial intensity distribution (RID)

The radial intensity distribution (RID) describes how a signal is spatially distributed from an objects' boundary (here the membrane) towards its centroid. The RID is determined by successively eroding the object boundaries and measuring the intensity in the resulting area. The intensity in the eroded layer can be easily calculated by subtracting signal intensities measured before and after the erosion step. Normalization to the total signal intensity provides the relative intensity within the eroded layer (see **equation 3** in the main text). As individual cells have different cellular dimensions, RID analysis requires a variable number of erosion steps. The number of erosion steps or absolute cell width can thus not be used as x-coordinate in RID plots. To circumvent this limitation, we measured the object area before each erosion step and normalized it to the original object area. As objects are eroded radially, the relative cell area correlates with cell width and can be used as approximation for the distance to the cell centre.

Supplementary Note 3: Differences between *X. doucetiae* and *E. coli*

Xenorhabus doucetiae is a gram-negative bacterium that lives in symbiosis with the entomopathogenic nematode *Steinernema diaprepesi*. Although it is not found in the free-living form in nature, *X. doucetiae* can be grown in the laboratory under ambient conditions (e.g. LB Lennox, 30°C, with agitation). Its genome size is ~ 4.2 Mbp and it harbours a single plasmid with ~ 8.5 kbp. However, and in contrast to *E. coli*, a large fraction of genomic DNA is dedicated to the production of natural products, which are mostly produced in stationary phase. During exponential phase, the growth rate is of *X. doucetiae* is slightly lower than that of *E. coli* (~35 min compared to 27 min in our experiments). However, *X. doucetiae* cells have a larger volume during exponential growth in rich medium. We determined the cell diameter to be 1.42 ± 0.12 µm for the MreB^{sw}-sfGFP fusion strain. As the larger cell diameter results in a smaller influence of projection (see Supplementary Note 1), we considered *X. doucetiae* to be an interesting additional target for our study. Notably, *E. coli* and *X. doucetiae* share homologues of most major nucleoid associated proteins (NAPs) (FIS, H-NS, HupA, HupB, IHF, SlmA, Dps, MukB), except for StpA. This indicates that the effect of NAPs on nucleoid structure might be comparable.

Supplementary Figures

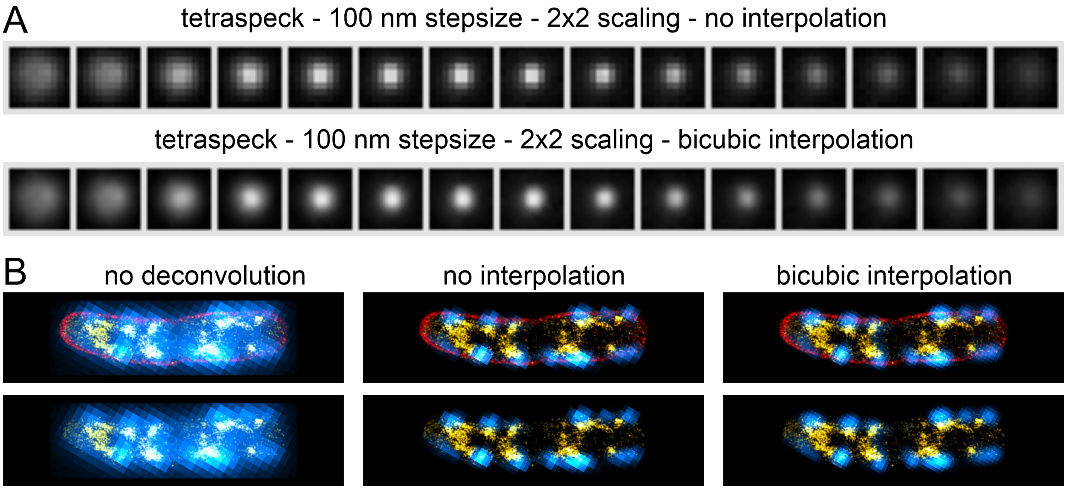

Figure S1: Deconvolution of MreB-sfGFP^{sw} signal. [A] Representative stack of an individual bead (100 nm tetraspeck), 2x scaled without and with interpolation (bicubic). [B] Exemplary E. coli NO34 cell showing the effect of deconvolution using the Fiji plugin "3D iterative deconvolution" (see methods). 3-4 center planes of the MreB-sfGFP^{sw} stack (cyan hot) were averaged and overlaid with the DNA (yellow hot) and membrane (red) PAINT signal. Deconvolution clearly enhances resolution and contrast of the MreB signal.

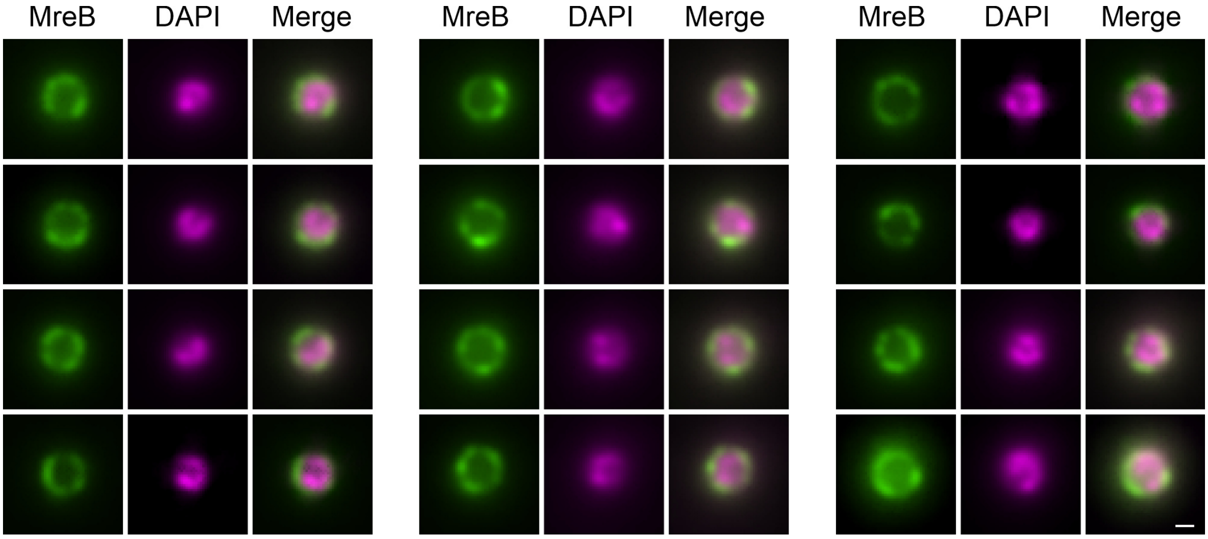

Figure S2: VerCINI measurements of DAPI-stained NO34 cells. Cross-sections of individual vertical cells expressing MreB-sfGFP^{sw} (green) and stained with DAPI (magenta). Scale bar is 0.5 µm.

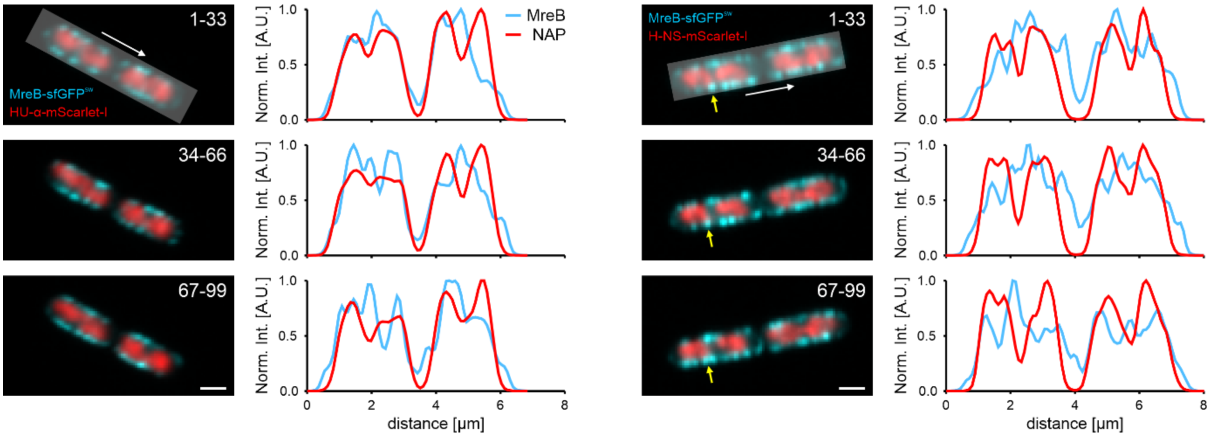

Figure S3: Representative confocal images of live E. coli cells expressing MreB-sfGFP (cyan) and HU-α-mScarlet-I (left panel, red) or H-NS-mScarlet-I (right panel, red). A subset of 33 frames were averaged and intensities were plotted along the bacterial long axis (white arrow and shaded area). Yellow arrow highlights an elongasome that stays close to a nucleoid filament over the entire time course of imaging. Scale bar is 1 µm. Source data are provided as a Source Data file.

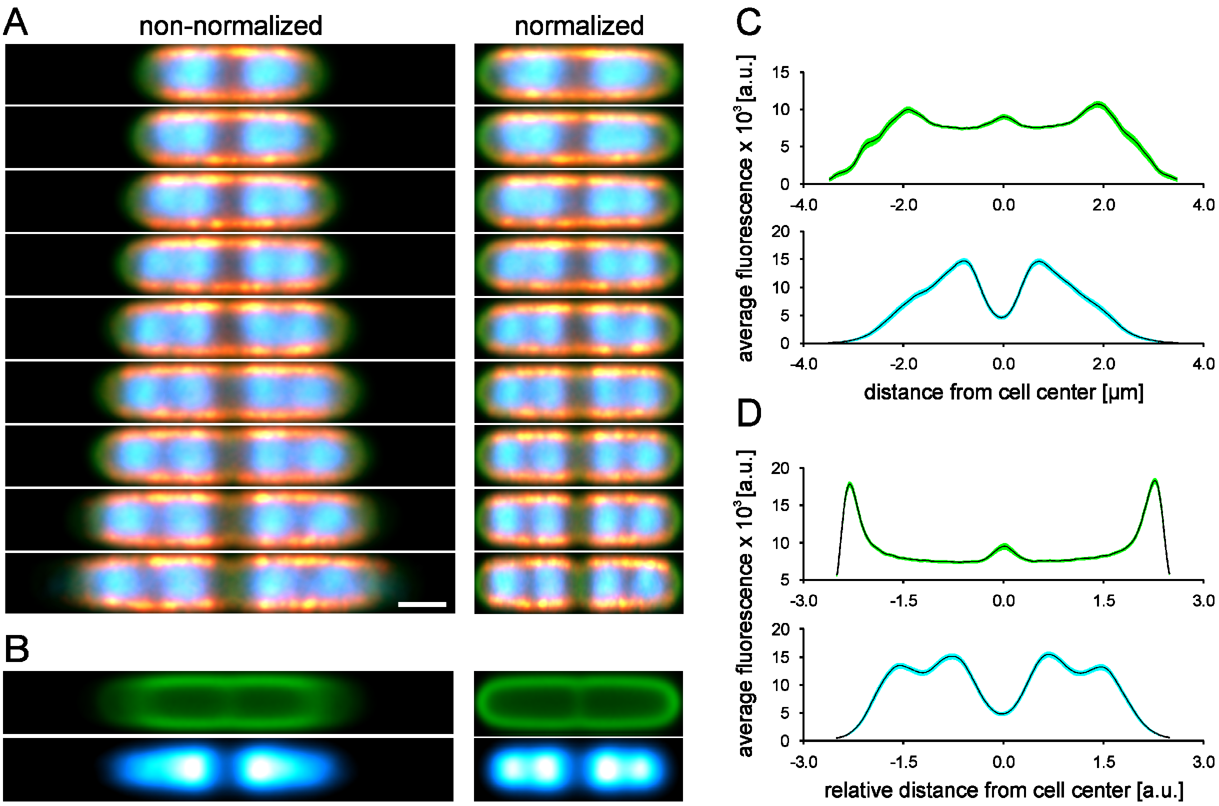

Figure S4: Averaging of non-normalized and normalized confocal images. [A] Average images of individual E. coli NO34 cells (N = 157 cells) of varying length. 40 cells were averaged for each image with 20 cells overlap. MreB-sfGFP^{sw} is shown in red hot, membrane in green and the nucleoid in cyan hot. Color-code was changed for visualization purpose. [B] Average images of all cells (membrane and nucleoid), either non-normalized (left) or normalized (right). [C] Intensity plot along the length axis of non-normalized average image shown in [B]. [D] Intensity plot along the length axis of normalized average image shown in [B]. The bilobed structure of the sister chromosomes is visible in contrast to the plot in [C]. Scale bar in [A] is 1 µm.

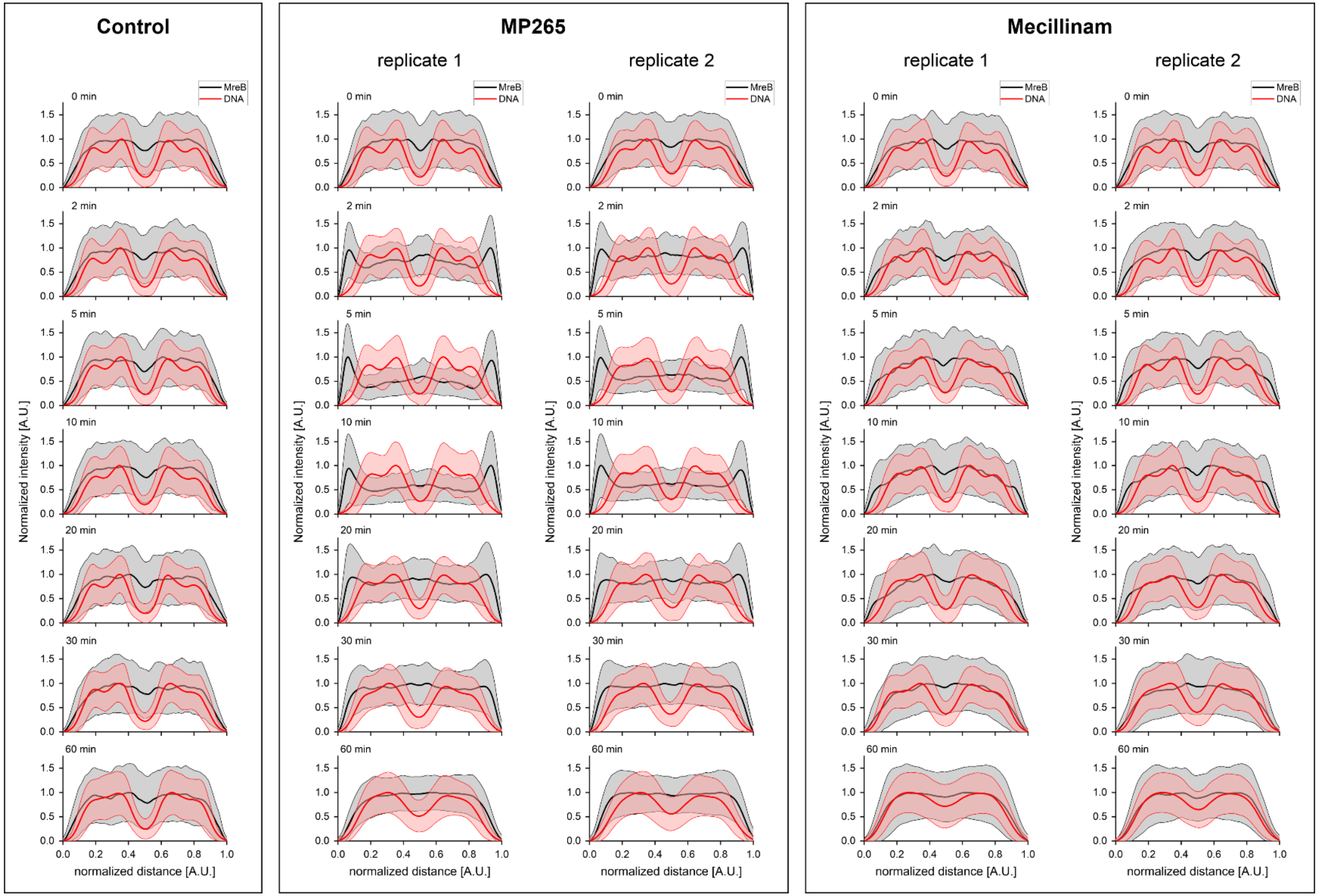

Figure S5: Length axis plots of MreB and DNA signal during perturbation of cell wall synthesis. Intensities were measured in confocal average and standard deviation images. Lines indicate the mean intensities and shaded areas the standard deviation. Cell numbers for each condition and time point are listed in Table S3. Source data is available under https://zenodo.org/records/14967865.

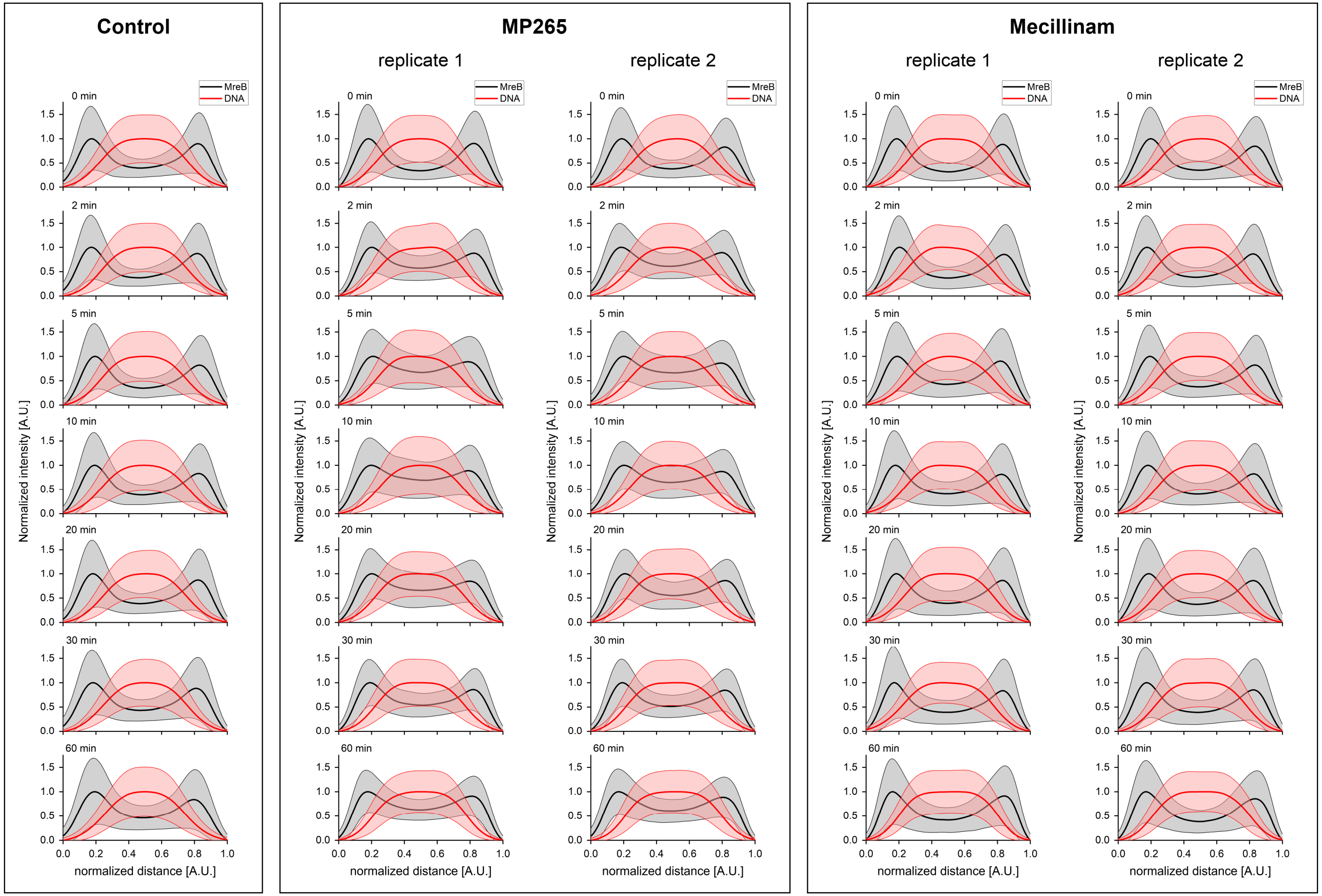

Figure S6: Cross axis plots of MreB and DNA signal during perturbation of cell wall synthesis. Intensities were measured in confocal average and standard deviation images. Lines indicate the mean intensities and shaded areas the standard deviation. Cell numbers for each condition and time point are listed in Table S3. Source data is available under https://zenodo.org/records/14967865.

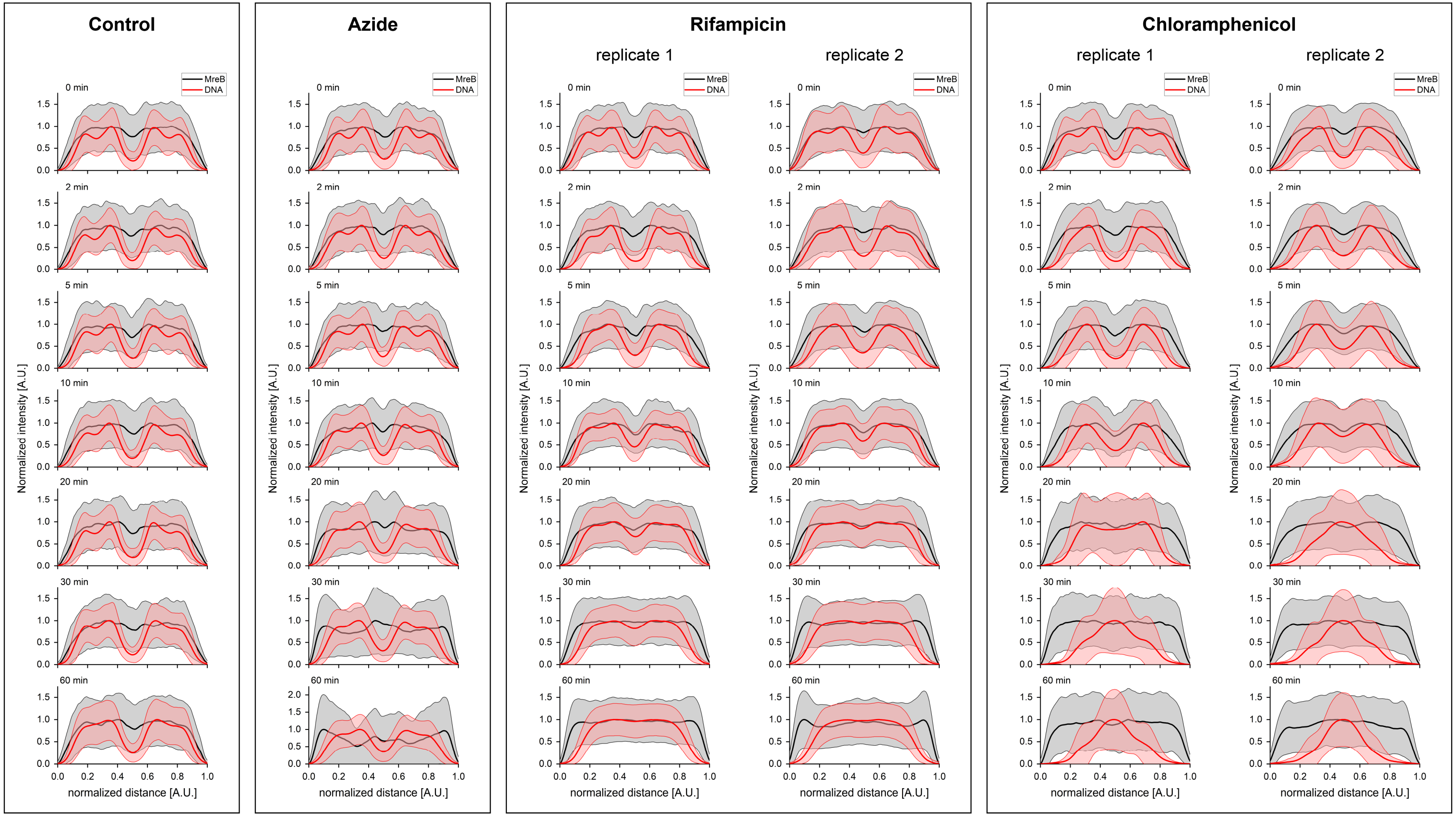

Figure S7: Length axis plots of MreB and DNA signal during perturbation of protein biosynthesis and transport. Intensities were measured in confocal average and standard deviation images. Lines indicate the mean intensities and shaded areas the standard deviation. Cell numbers for each condition and time point are listed in Table S3. Source data is available under https://zenodo.org/records/14967865.

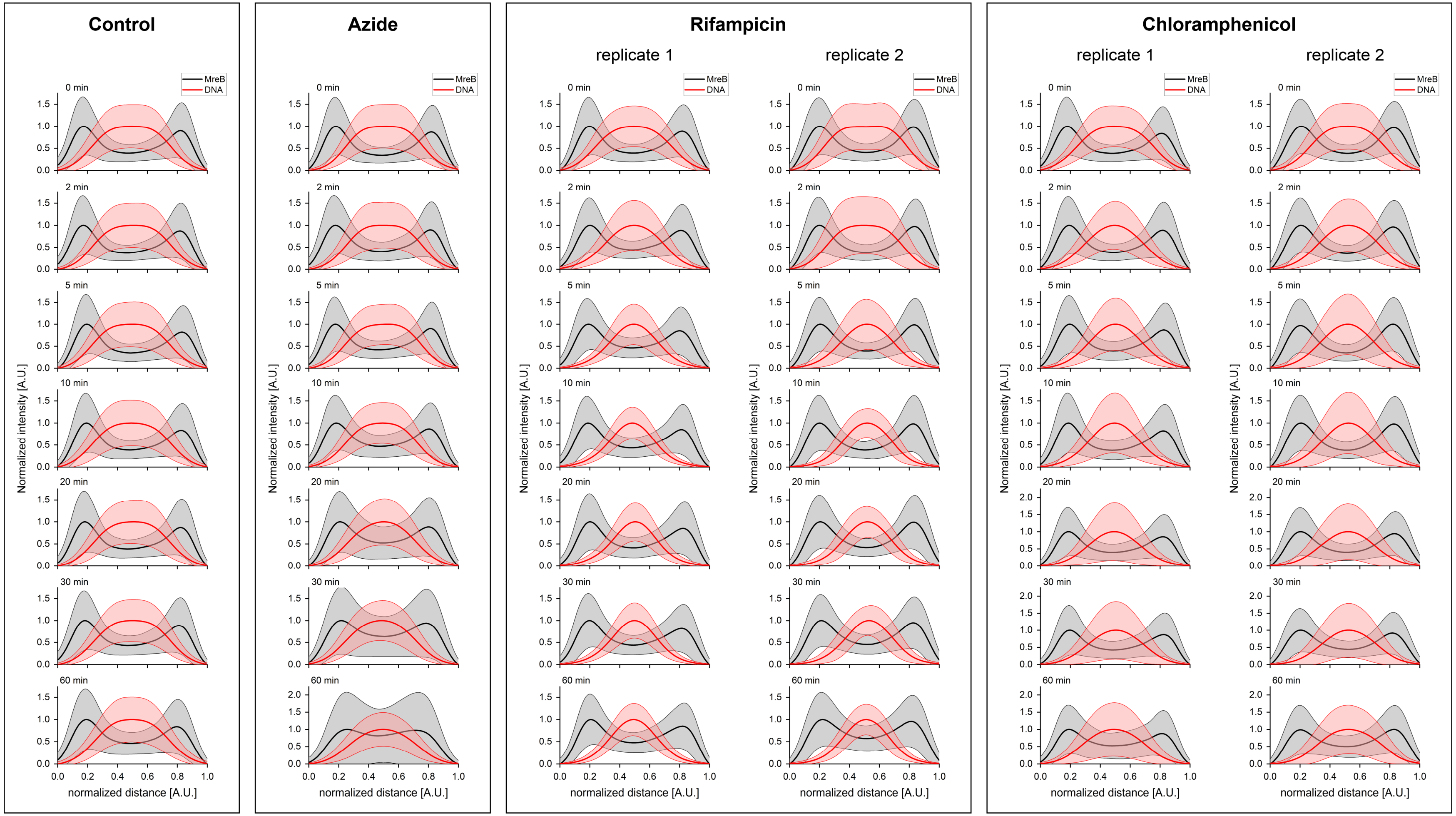

Figure S8: Cross axis plots of MreB and DNA signal during perturbation of protein biosynthesis and transport. Intensities were measured in confocal average and standard deviation images. Lines indicate the mean intensities and shaded areas the standard deviation. Cell numbers for each condition and time point are listed in Table S3. Source data is available under https://zenodo.org/records/14967865.

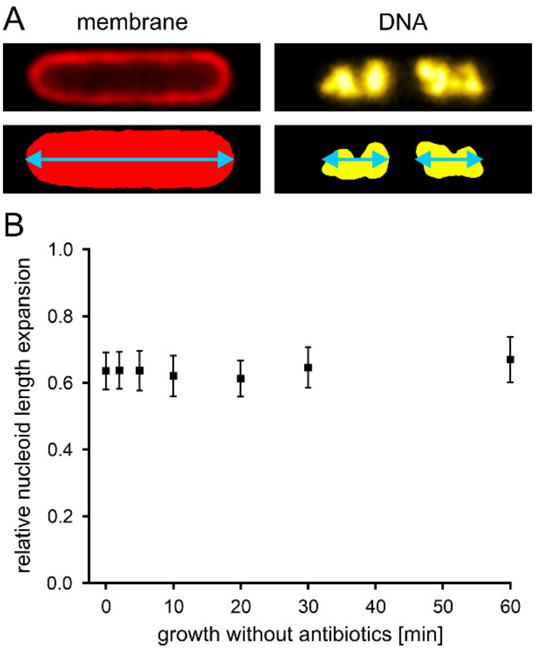

Figure S9: Measurement of relative nucleoid length of untreated cells over time. [A] Representative NO34 cell labeled for membrane (Nile red, red) and DNA (DAPI, yellow hot). Signal is binarized and cell (left) and nucleoid (right) lengths are measured using a custom-written Fiji macro. [B] Relative nucleoid length over time. Aliquots of a control culture were fixed at the indicated time points and imaged using confocal microscopy. The average relative nucleoid length does not change with increasing culture density during the observation period. Data points represent mean values and error bars the standard deviation. N = 213, 161, 154, 165, 121, 146 and 153 cells for 0, 2, 5, 10, 20, 30 and 60 min timepoints, extracted from a single biological replicate. Source data are provided as a Source Data file.

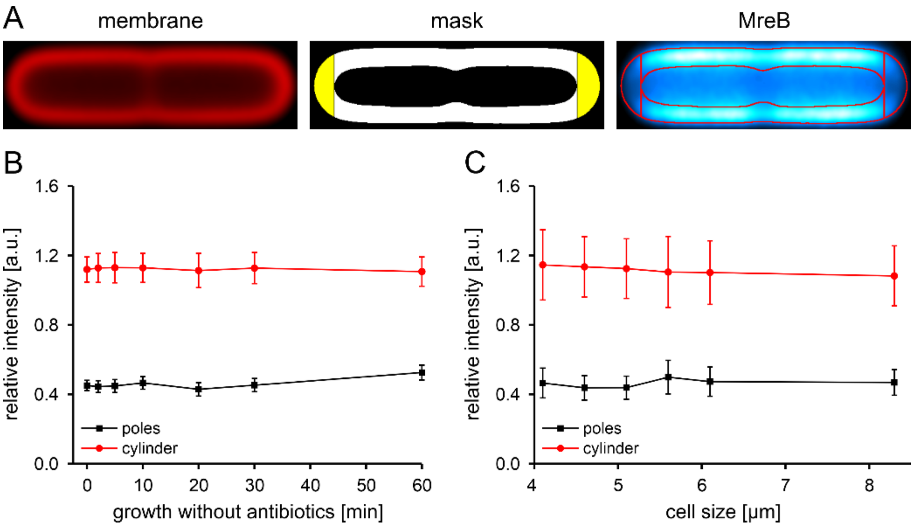

Figure S10: Analysis of the MreB intensity distribution along the bacterial membrane in confocal average images. [A] Schematic of the analysis. The average membrane image is thresholded using Otsu's method. Poles (mid panel, yellow areas) are segmented according to the cytosol (black area). Pole (yellow areas) and cylindrical membrane sections (white areas) are used to measure the MreB-sfGFP^{sw} intensity in the MreB average image (right panel). [B] Relative MreB intensity over time in an untreated culture. MreB distribution remains constant over the entire time course. N = 213, 161, 154, 165, 121, 146 and 153 cells for 0, 2, 5, 10, 20, 30 and 60 min timepoints extracted from a single biological replicate. [C] Length-dependence of the MreB distribution. A dataset (untreated culture, t = 0 min) was split into subsets according to cell length (0.5 µm interval except > 6 µm) and average images were calculated. MreB intensity distribution is insensitive to cell length. Higher errors are a result of the lower cell count used for generation of the average images. Data points represent individual values and error bars the standard error of the mean calculated from standard deviation images of the averaging process. Source data are provided as a Source Data file.

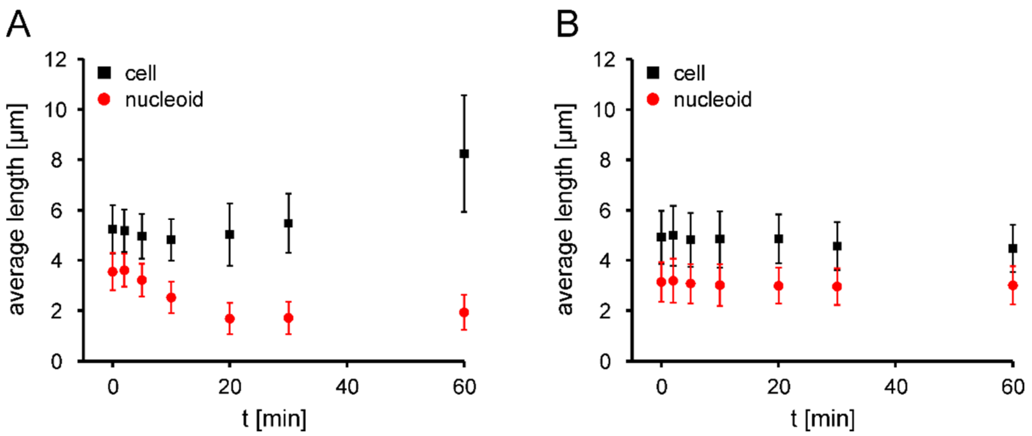

Figure S11: Measurement of cell and nucleoid lengths during nalidixate treatment. [A] Average length over time during exposure to nalidixate. Cell length (black) increases with nalidixate exposure, while nucleoid length (red) shrinks until it reaches a plateau after 20 min. [B] Average length over time in an untreated control culture. Average cell and nucleoid lengths remain constant during the observation period. Data points represent mean values and error bars the respective standard deviation. N = 182, 121, 154, 105, 137, 98 and 90 cells for 0, 2, 5, 10, 20, 30 and 60 min timepoints, extracted from a single biological replicate. Source data are provided as a Source Data file.

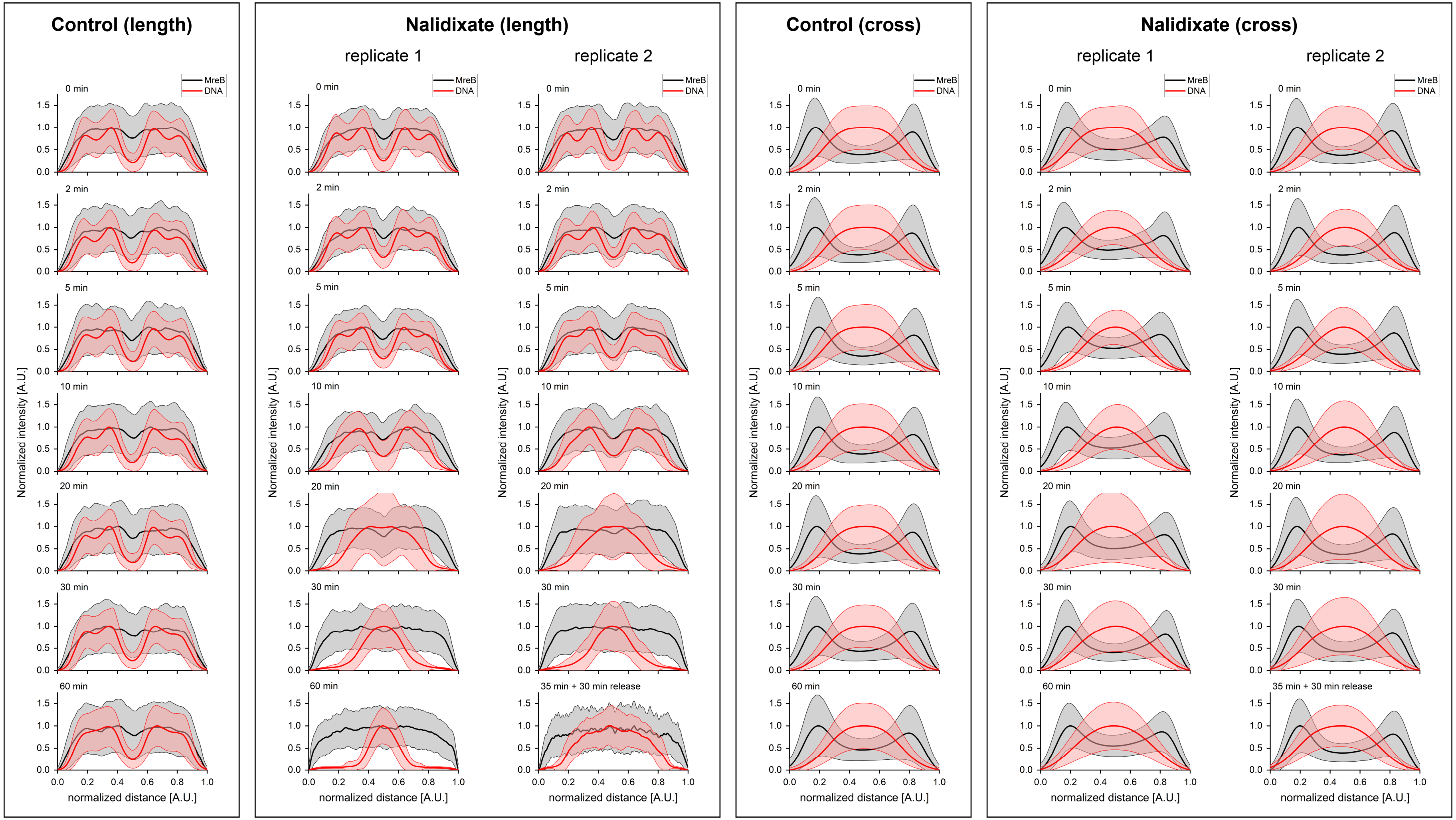

Figure S12: Length and cross axis plots of MreB and DNA signal inhibition of DNA replication. Intensities were measured in confocal average and standard deviation images. Lines indicate the mean intensities and shaded areas the standard deviation. Cell numbers for each condition and time point are listed in Table S3. Source data is available under https://zenodo.org/records/14967865.

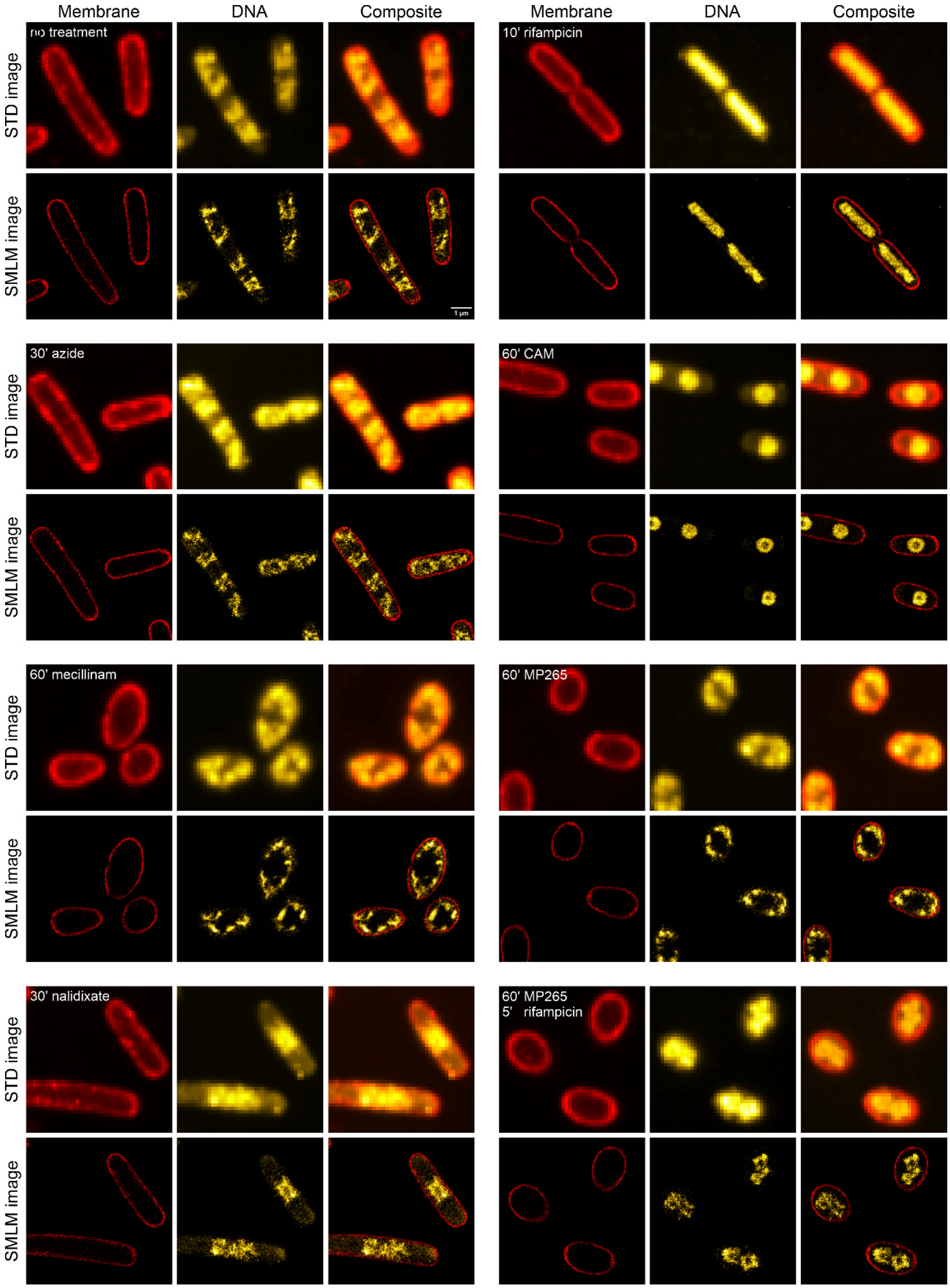

Figure S13: Comparison of diffraction-limited standard deviation images with their corresponding super-resolved PAINT images. PAINT images reveal detailed features of the membrane (red) and nucleoid (yellow), which are not resolvable in the standard deviation image. Scale bar is 1 µm.

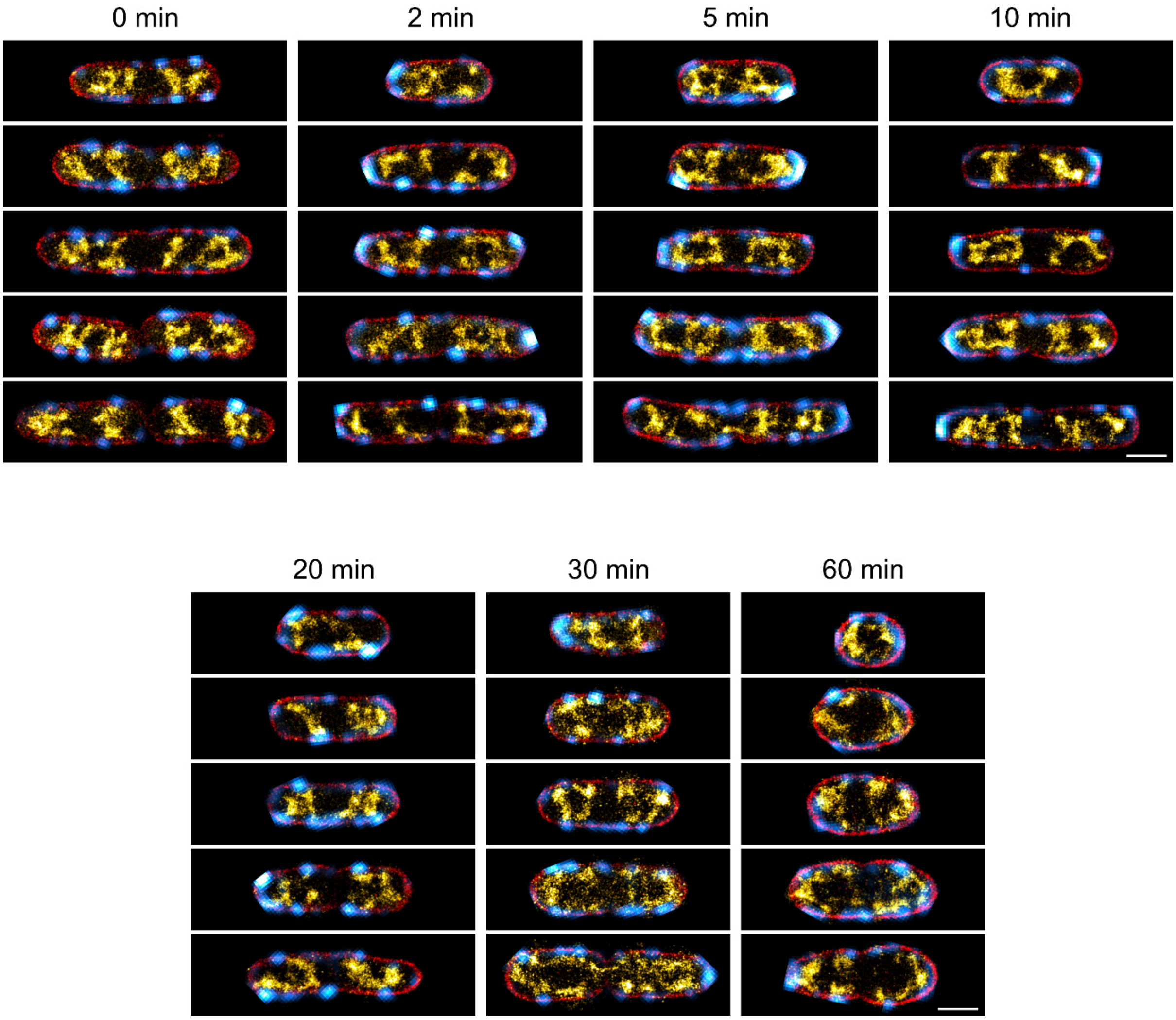

Figure S14: Exemplary dual-color PAINT images of fixed E. coli NO34 cells treated with 25 µM MP265. MreB-sfGFP^{sw} is shown in cyan hot, membrane in red and DNA in yellow hot. Scale bars are 1 µm.

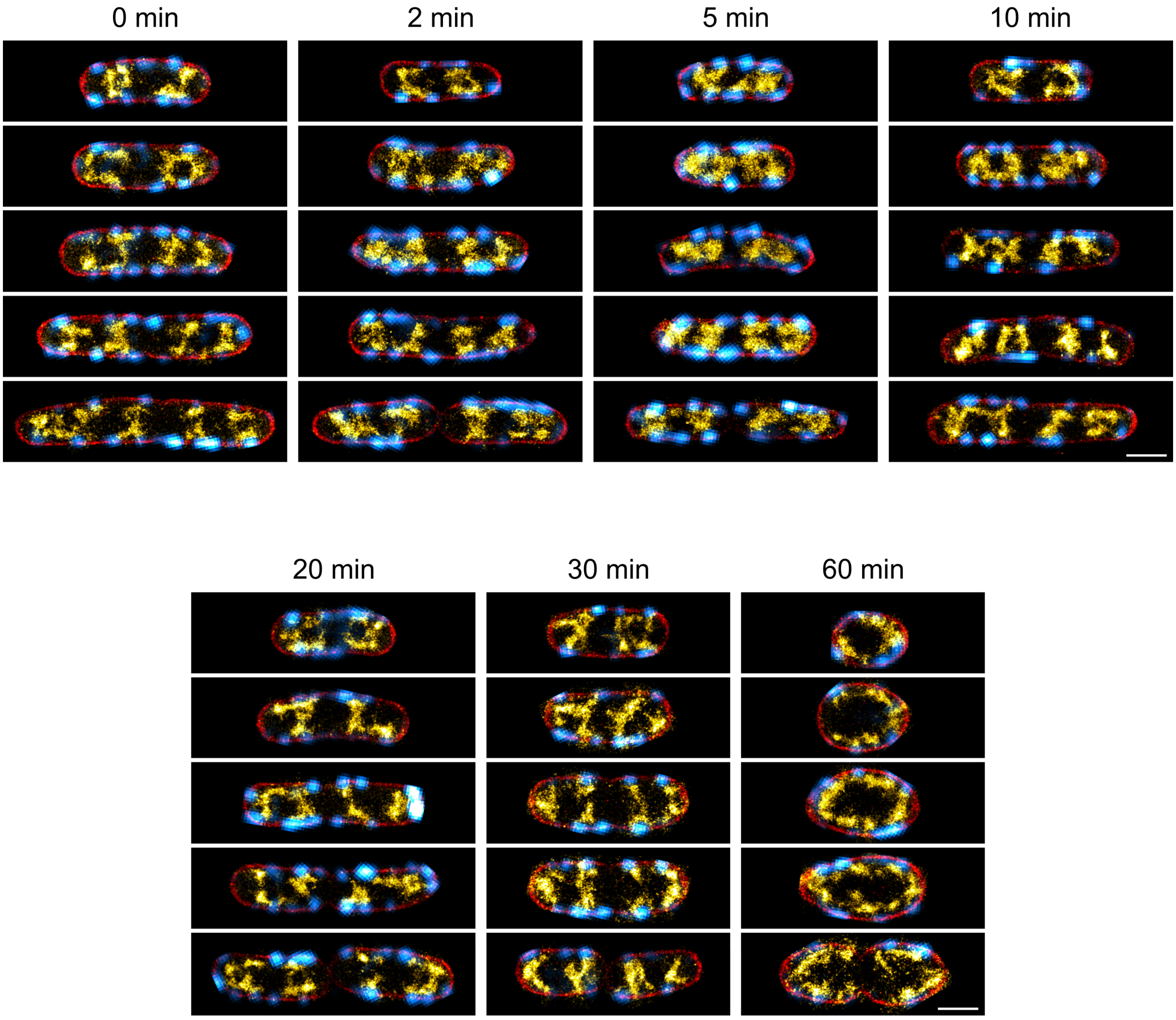

Figure S15: Exemplary dual-color PAINT images of fixed E. coli NO34 cells treated with 2 µg/ml Mecillinam. MreB-sfGFP^{sw} is shown in cyan hot, membrane in red and DNA in yellow hot. Scale bars are 1 µm.

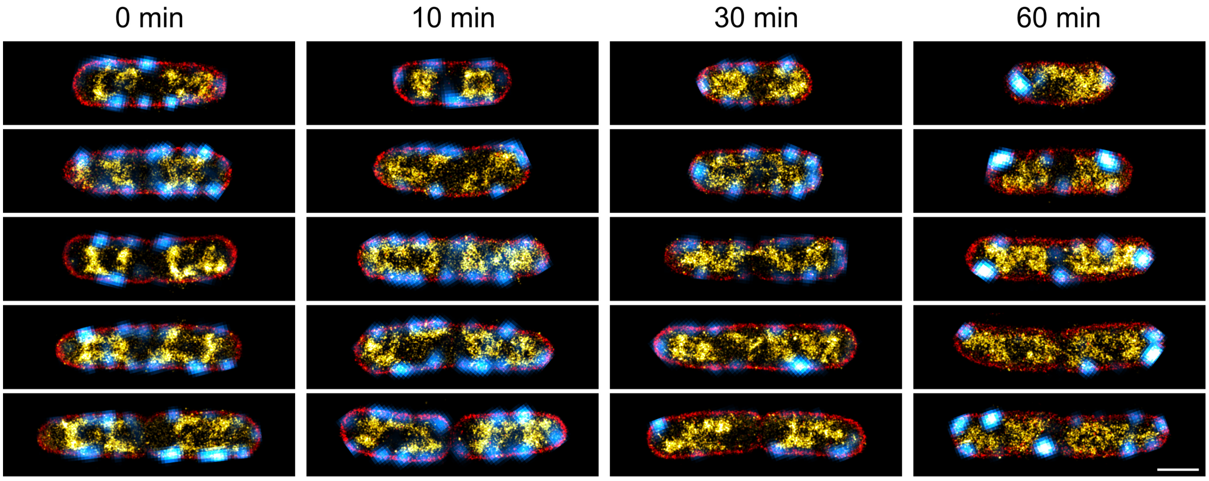

Figure S16: Exemplary dual-color PAINT images of fixed E. coli NO34 cells treated with 1 µM sodium azide (NaN_3). MreB-sfGFP^{sw} is shown in cyan hot, membrane in red and DNA in yellow hot. Scale bars are 1 µm.

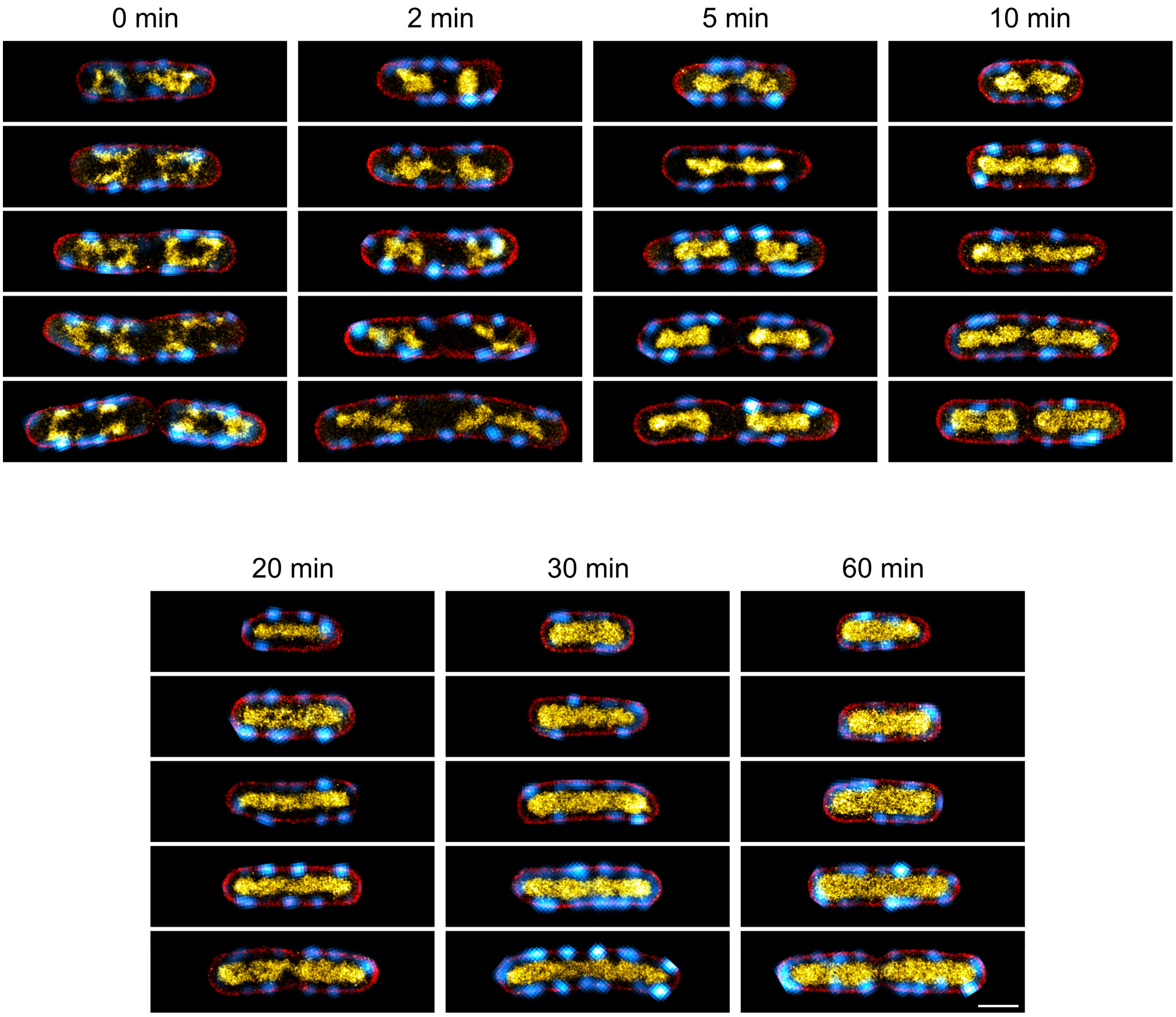

Figure S17: Exemplary dual-color PAINT images of fixed E. coli NO34 cells treated with 100 µg/ml rifampicin. MreB-sfGFP^{sw} is shown in cyan hot, membrane in red and DNA in yellow hot. Scale bars are 1 µm.

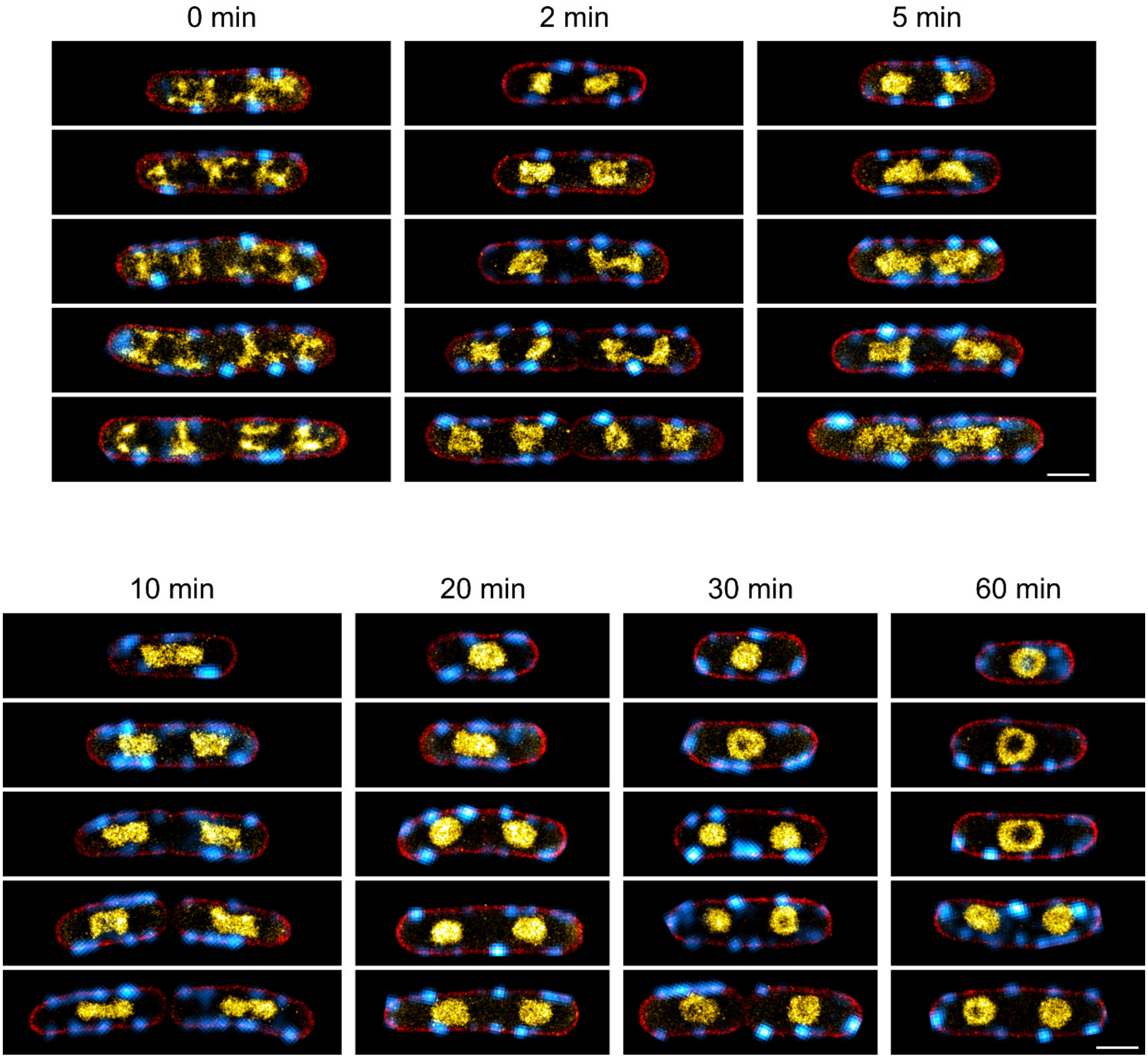

Figure S18: Exemplary dual-color PAINT images of fixed E. coli NO34 cells treated with 50 µg/ml chloramphenicol. MreB-sfGFP^{sw} is shown in cyan hot, membrane in red and DNA in yellow hot. Scale bars are 1 µm.

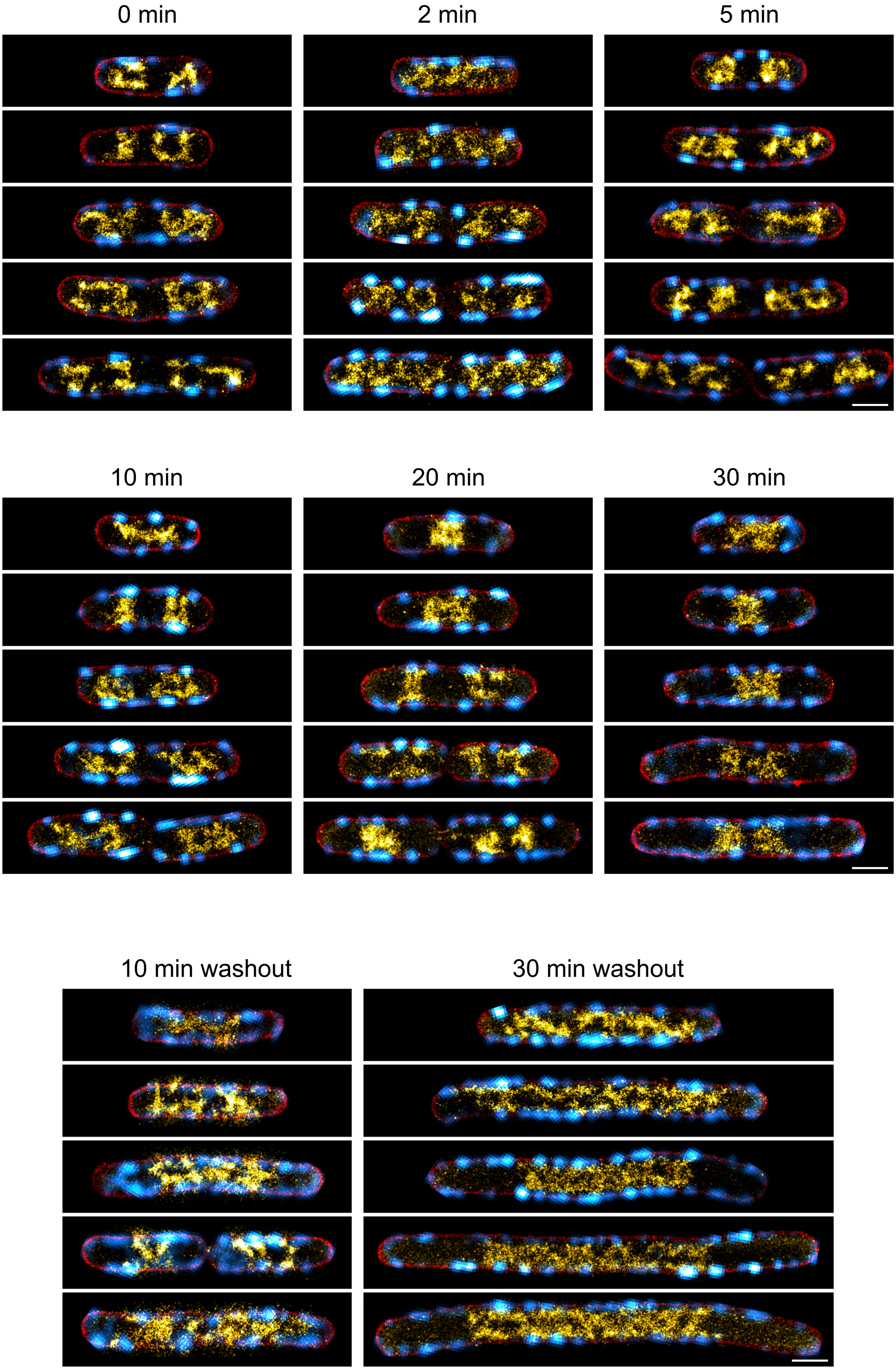

Figure S19: Exemplary dual-color PAINT images of fixed E. coli NO34 cells treated with 100 µg/ml nalidixate. MreB-sfGFP^{sw} is shown in cyan hot, membrane in red and DNA in yellow hot. Scale bars are 1 µm.

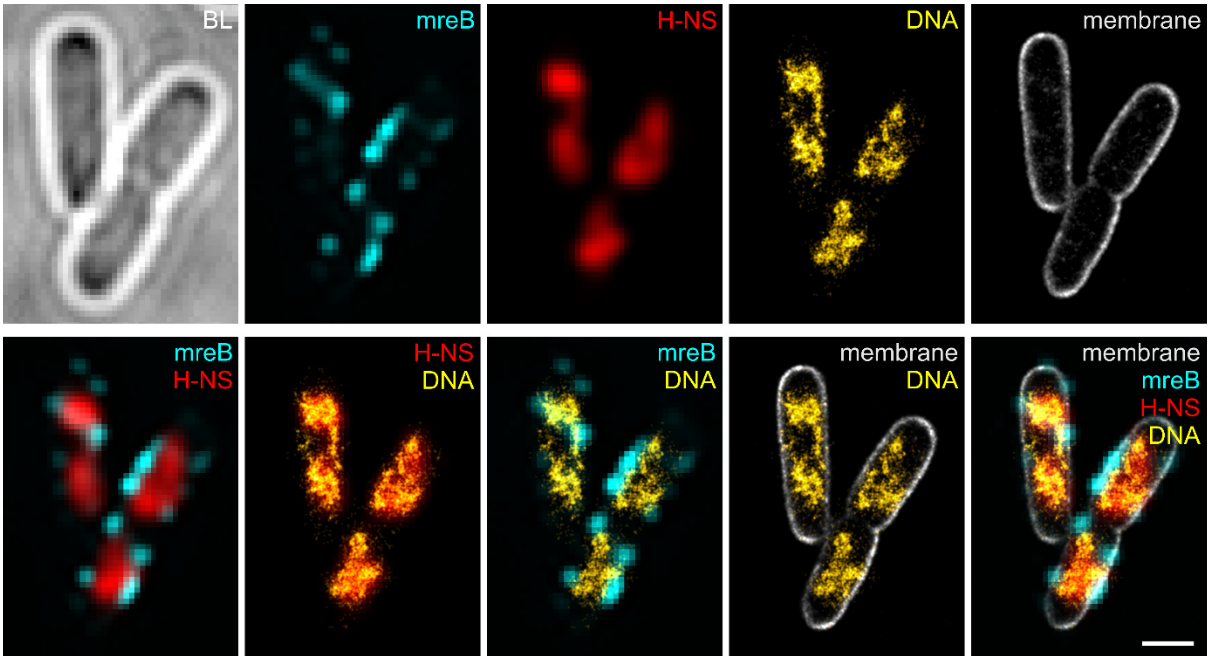

Figure S20: Dual-color PAINT imaging of fixed E. coli expressing MreB-sfGFP^{sw} and H-NS-mScarlet-I from the native locus. The images were acquired on a commercial N-STORM setup. Upper row shows the individual channels, while the lower row shows selected overlays. The super-resolution PAINT image matches the signal obtained from H-NS-mScarlet-I. Scale bar is 1 µm.

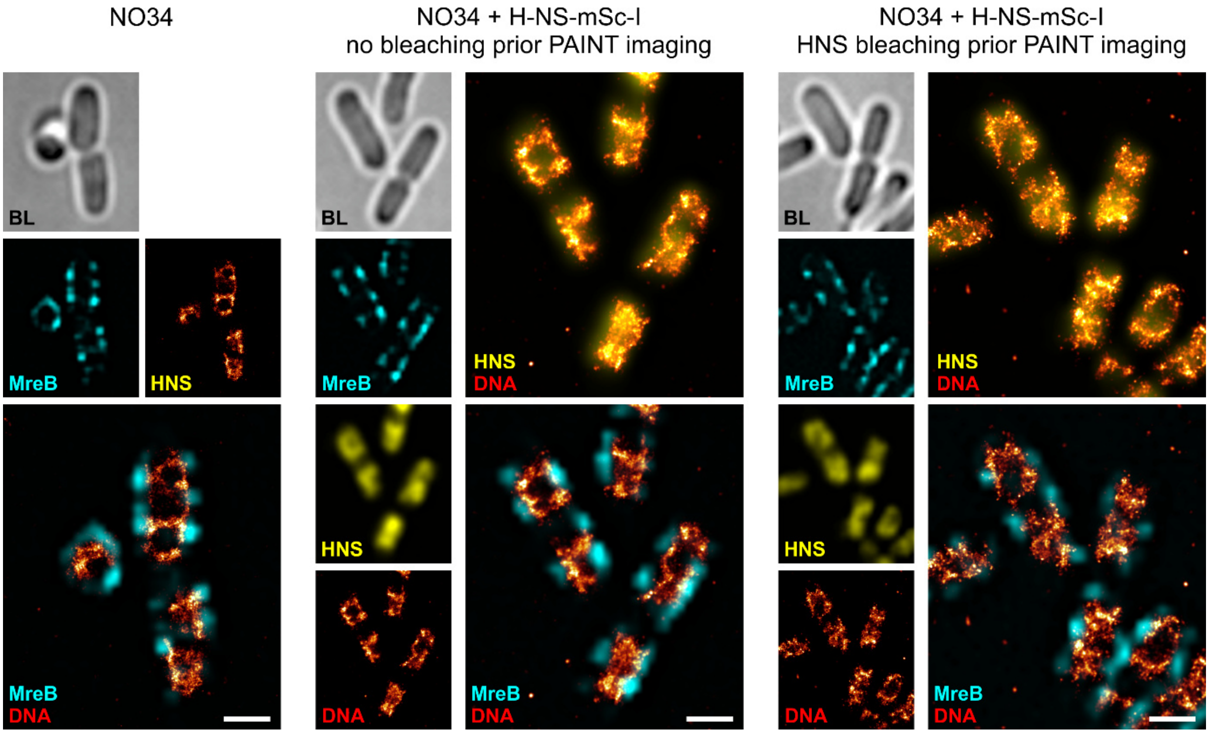

Figure S21: PAINT imaging of the nucleoid in fixed E. coli cells expressing MreB-sfGFP^{sw} and H-NS-mScarlet-I from the native locus. Images were acquired on a commercial Elyra PS1 setup. The super-resolution PAINT image matches the signal obtained from H-NS-mScarlet-I. Prebleaching H-NS-mScarlet-I was tested to reduce the background signal in the red channel (JF$_{646}$-Hoechst, DNA), which had no significant effect on the PAINT image quality. Scale bar is 1 µm.

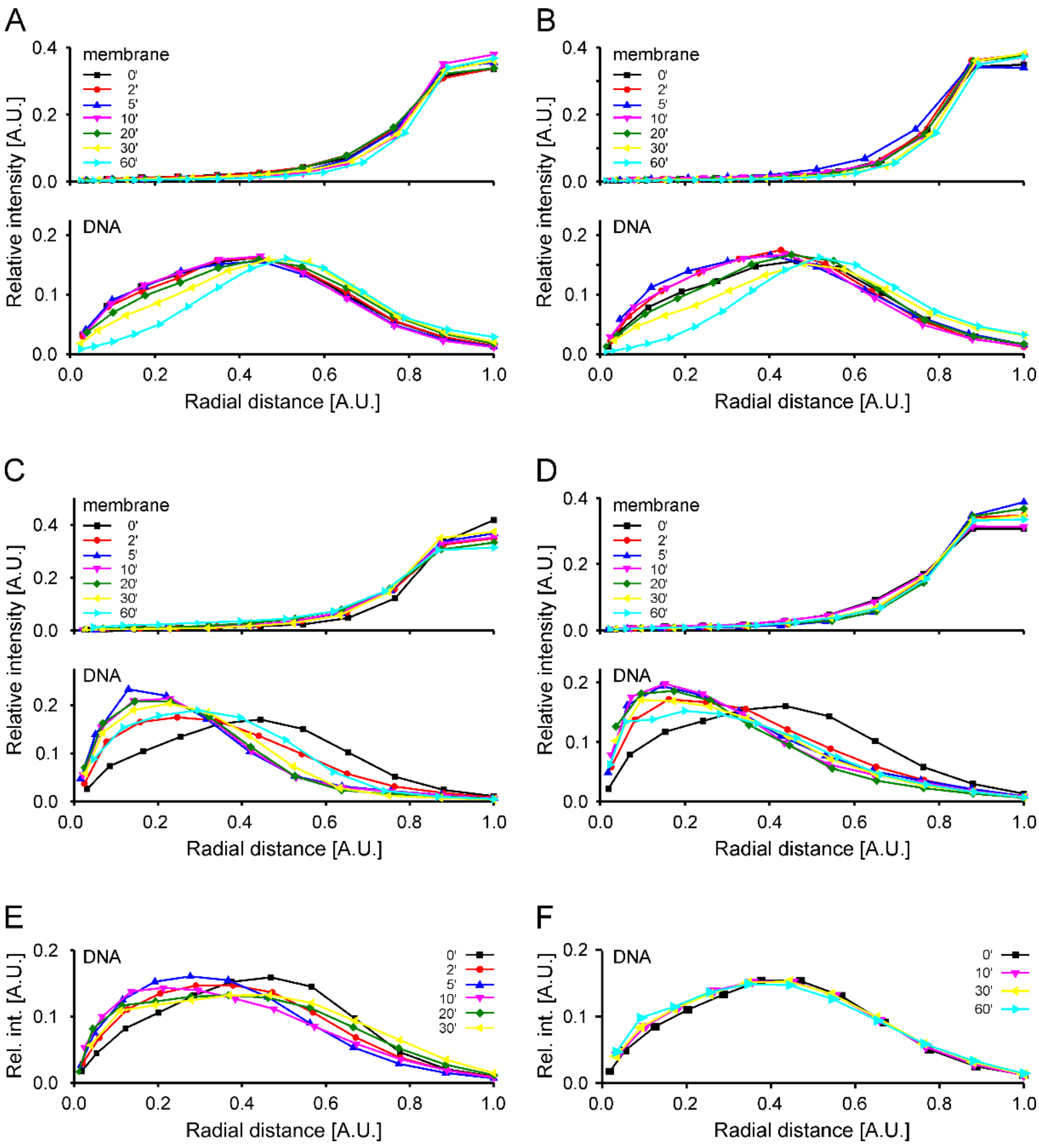

Figure S22: Erosion analysis plots of membrane and DNA signals for different drug treatments and time points. [A] MP265 treatment. [B] Mecillinam treatment. [C] Rifampicin treatment. [D] Chloramphenicol treatment. [E] DNA signal of cells treated with nalidixate. [F] DNA signal of cells treated with sodium azide. Values represent mean values. Error bars are omitted for visualization. Source data are provided as a Source Data file.

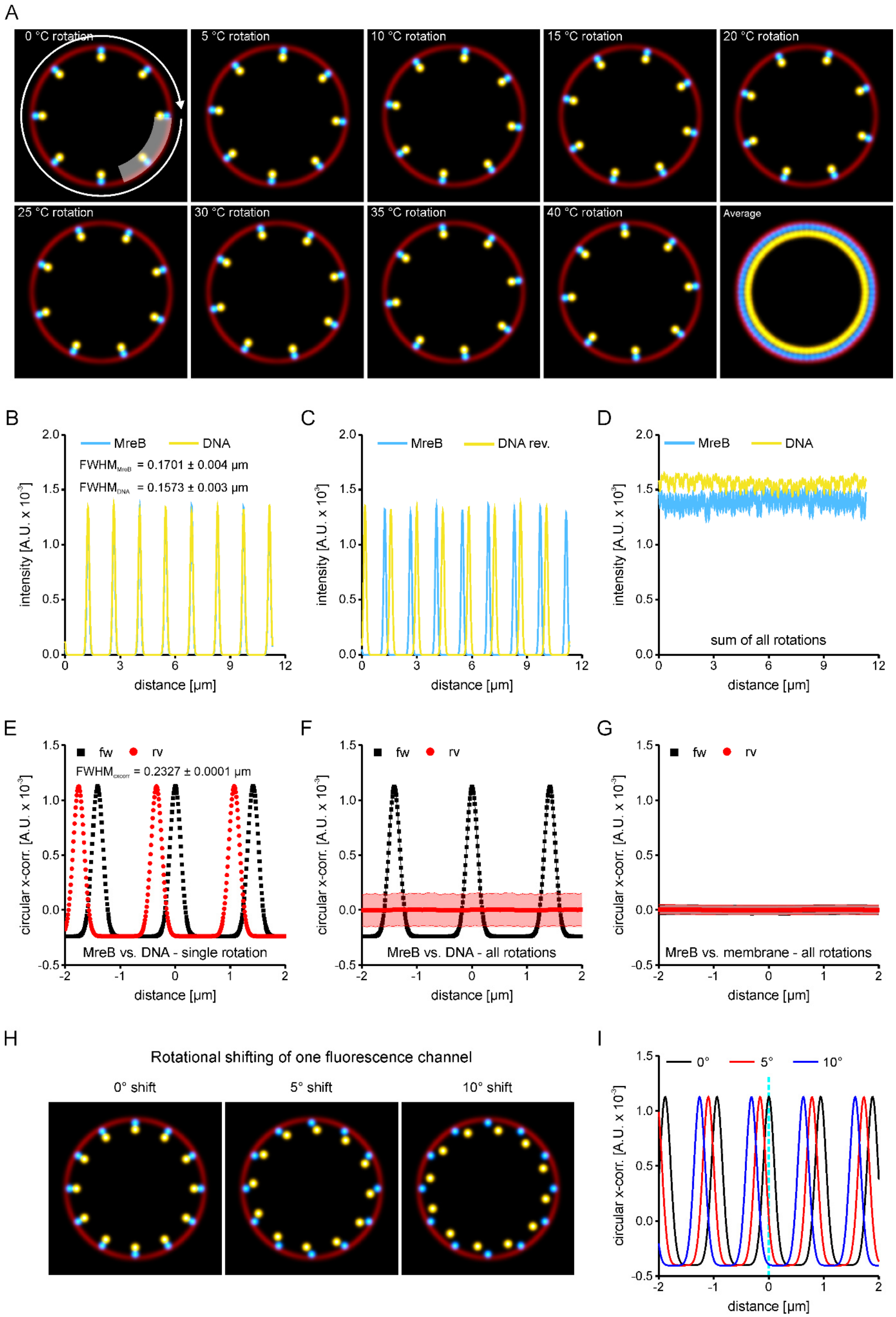

Figure S23: Circular cross correlation of simulated data. [A] 8 equidistantly spaced spots were positioned on a circle (mimicking the bacterial membranes) with displacement towards the center to mimic MreB and DNA. Heterogeneity is introduced by

rotating the spots with an increment of 5°. The average image shows the complete coverage of the circle area. The intensity trace is measured by a line plot of specific thickness along the circle perimeter (white arrow and shaded area). [B] Intensity trace for a single rotation in forward direction (same direction of MreB and DNA traces). [C] Intensity trace for a single rotation in reverse direction (DNA trace was inverted). The signal is shifted as the circular selection is opened at an arbitrary position. Thus, the inverted trace is shifted based on the starting point of the line plot. [D] Intensity traces of the average image shown in [A]. [E] Circular cross-correlation (x-corr) of a single rotation. For intensity traces aligned in the same direction, a peak is observed at the zero position. Neighboring peaks represent correlations to neighboring spots. The FWHM of 0.2327 hereby matches the convolution of the FWHM of both signals as shown in [B]. [F] Average circular x-corr of all rotations shown in [A]. The cross-correlations of the original intensity traces do not change, while they average out for MreB signals and the inverted DNA intensity traces. [G] Control analysis of the MreB signal vs the membrane signal. In both original and reverse mode, the analysis expectedly shows no correlation. [H] Simulation of images with shifted channels. DNA signal was shifted by 5° or 10°. [I] Circular cross-correlations of the images shown in H. The shift of the DNA channel leads to a shifted peak in the cross-correlation analysis.

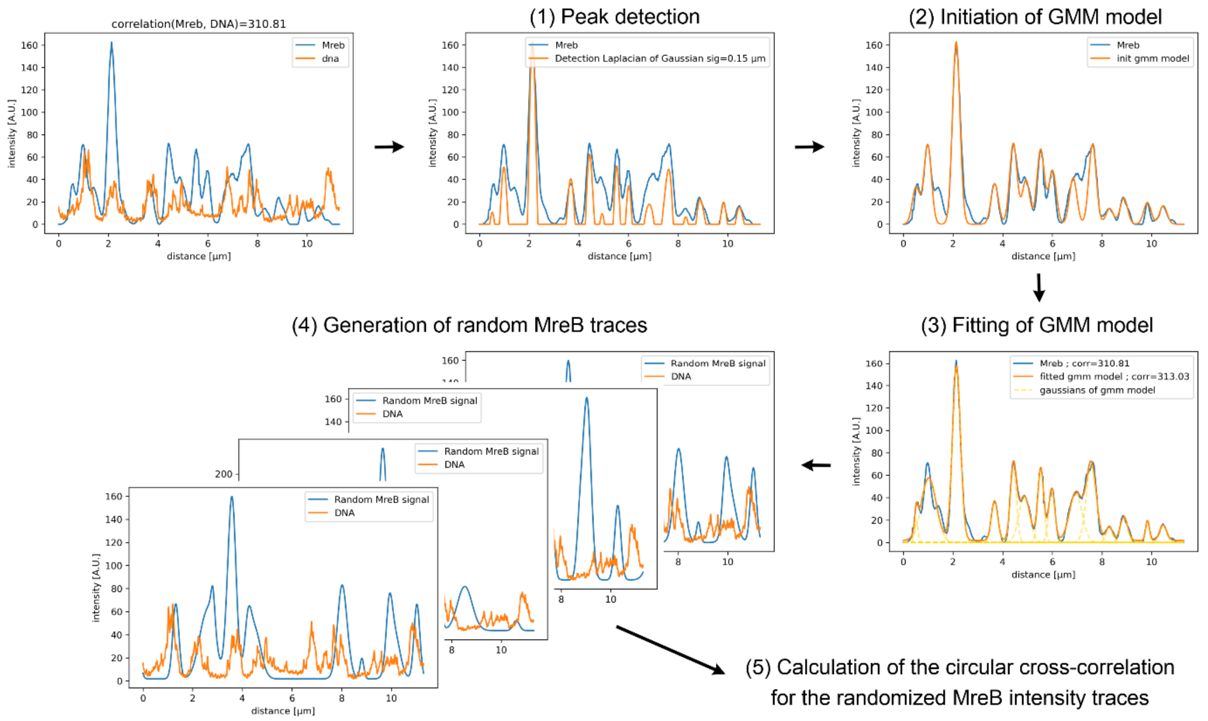

Figure S24: Data randomisation using a Gaussian Mixture Model (GMM). Shown are the intensity traces of the example bacteria shown in Figure 4. (1) First, peaks in the MreB intensity trace were detected using a Laplacian of Gaussian filter. (2) The GMM is initialized at the peak positions and fitted. The cross-correlation between the model and DNA intensity trace is calculated and compared to the cross-correlation of the input data. (4) Fitted peaks are shifted randomly to generate simulated MreB distributions.

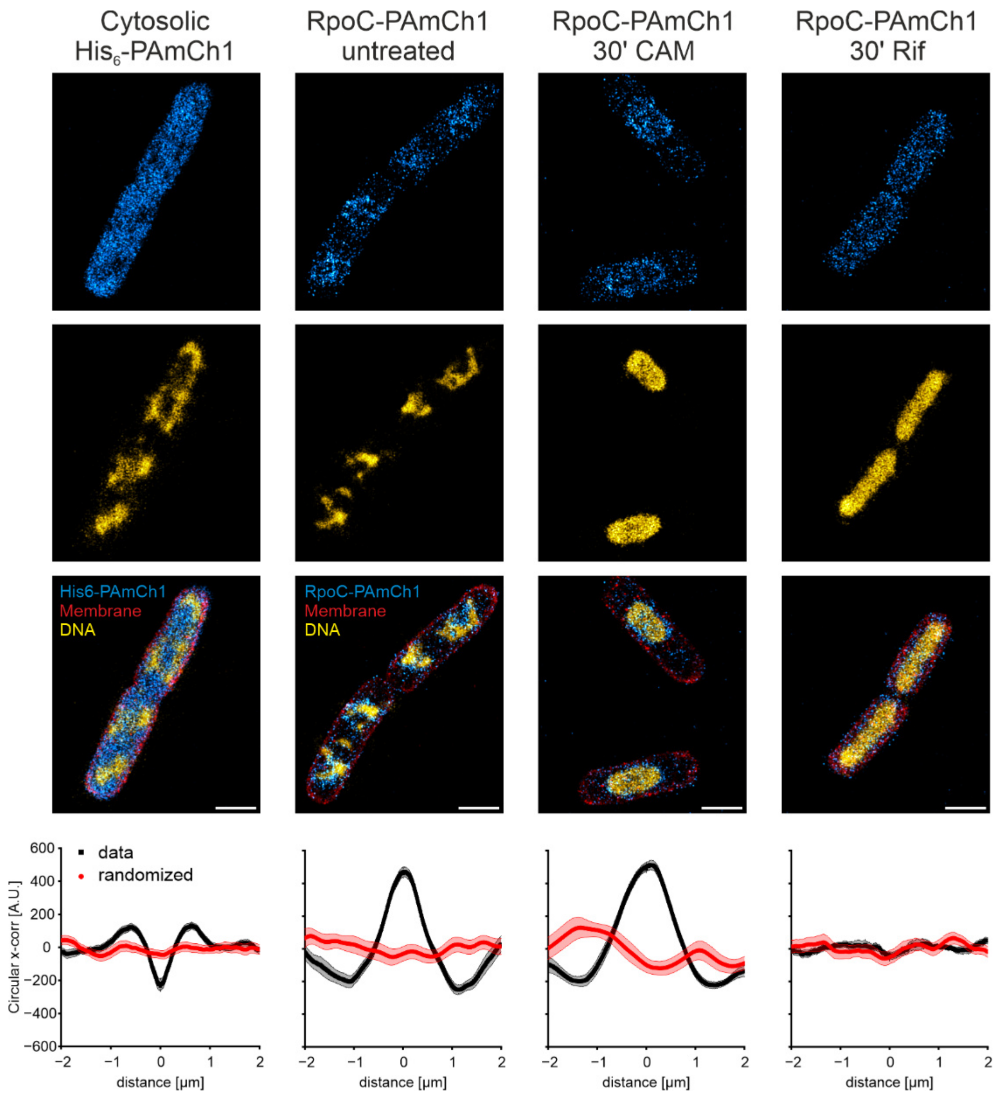

Figure S25: Circular cross-correlation of control multicolour SMLM measurements. Correlative PALM/PAINT super-resolution images of MG1655 strains either overexpressing His$_6$-PAmCh1 or expressing RpoC-PAmCh1 from the native chromosomal locus (KF26) were analysed using the presented cross-correlation approach. Anticorrelation is detected for cytosolic His$_6$-PAmCh1, which is excluded from the dense nucleoid region. A positive cross-correlation is detected for RpoC-PAmCh1 in untreated and chloramphenicol-treated cultures, in which the RNA polymerase is associated with the nucleoid. Dissociation of RNAP upon rifampicin treatment expectedly led to the loss of correlation. No cross-correlation is observed when the PAmCh1 signal is randomized using the GMM approach (Red curve and shaded areas; one simulation per cell). Data in the plots represents the mean value and shaded areas the corresponding SEM. N = 32 cells (cytosolic control), 17 cells (untreated KF26), 17 cells (chloramphenicol-treated KF26) and 18 cells (rifampicin-treated KF26). Scale bar is 1 µm. Source data are provided as a Source Data file.

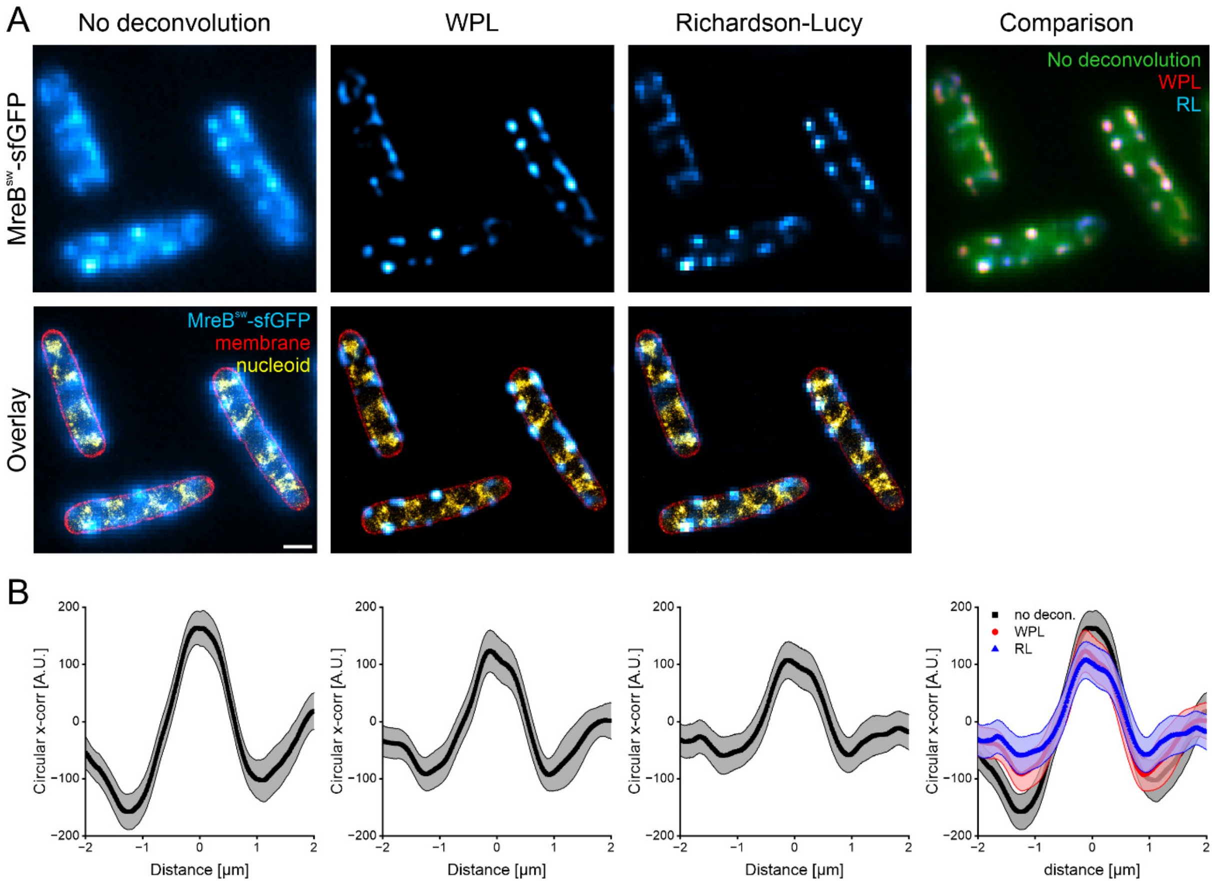

Figure S26: Comparison of deconvolution algorithms and effect on circular cross-correlation analysis. [A] Raw and deconvolved MreBs-sfGFP signal and overlay with DNA and membrane PAINT images. WPL = Wiener Filter Preconditioned Landweber, RL = Richardson-Lucy. [B] Circular cross-correlation analysis (N = 33 cells) for the overlay images shown in [A]. Source data are provided as a Source Data file.

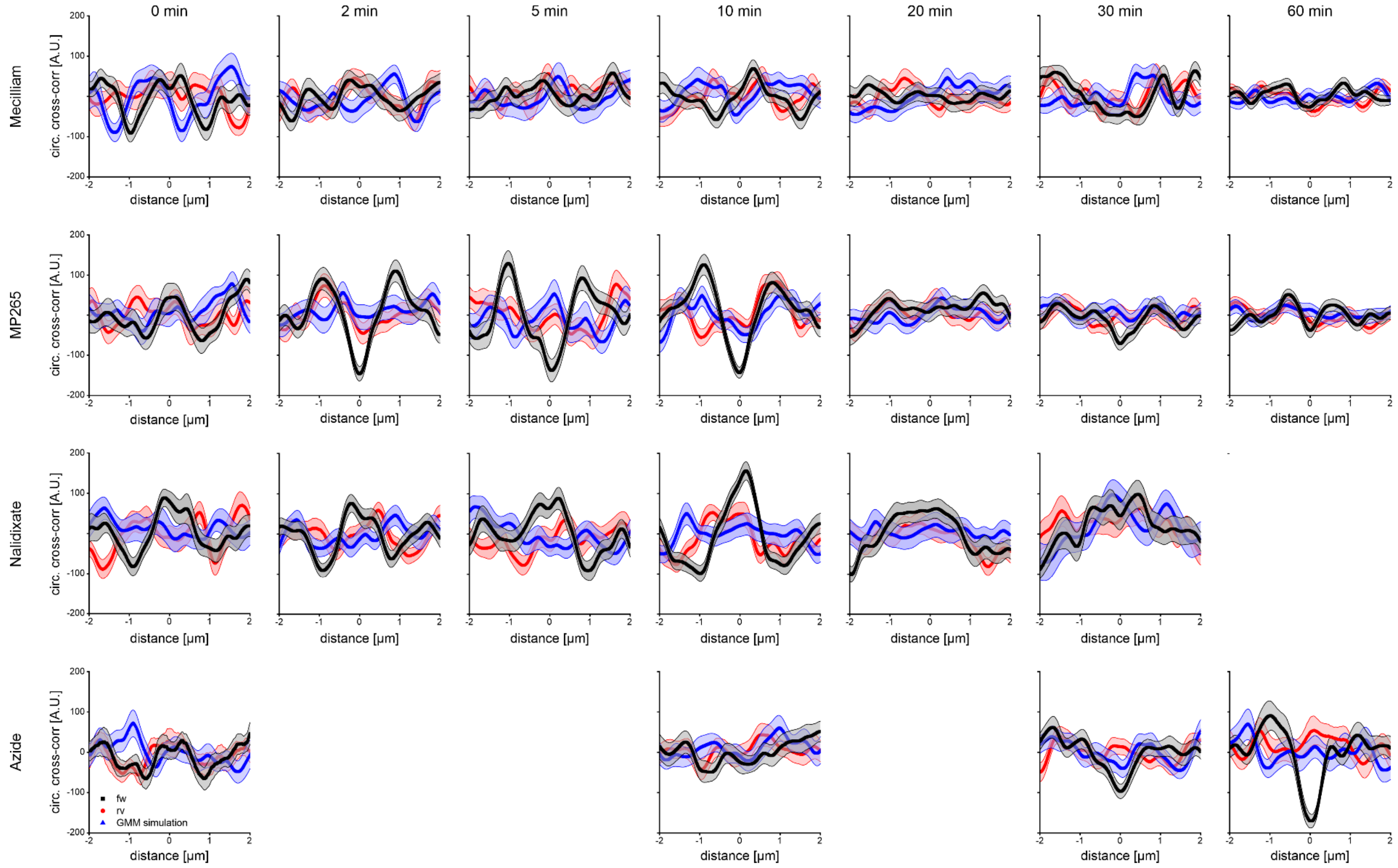

Figure S27: Circular x-corr analysis of different drug treatments. Correlations of original traces are shown in black, while correlation of MreB with inverted DNA signals is shown in red. The GMM randomization is shown in blue. Lines represent mean values und shaded areas the standard error of the mean. Source data are provided as a Source Data file.

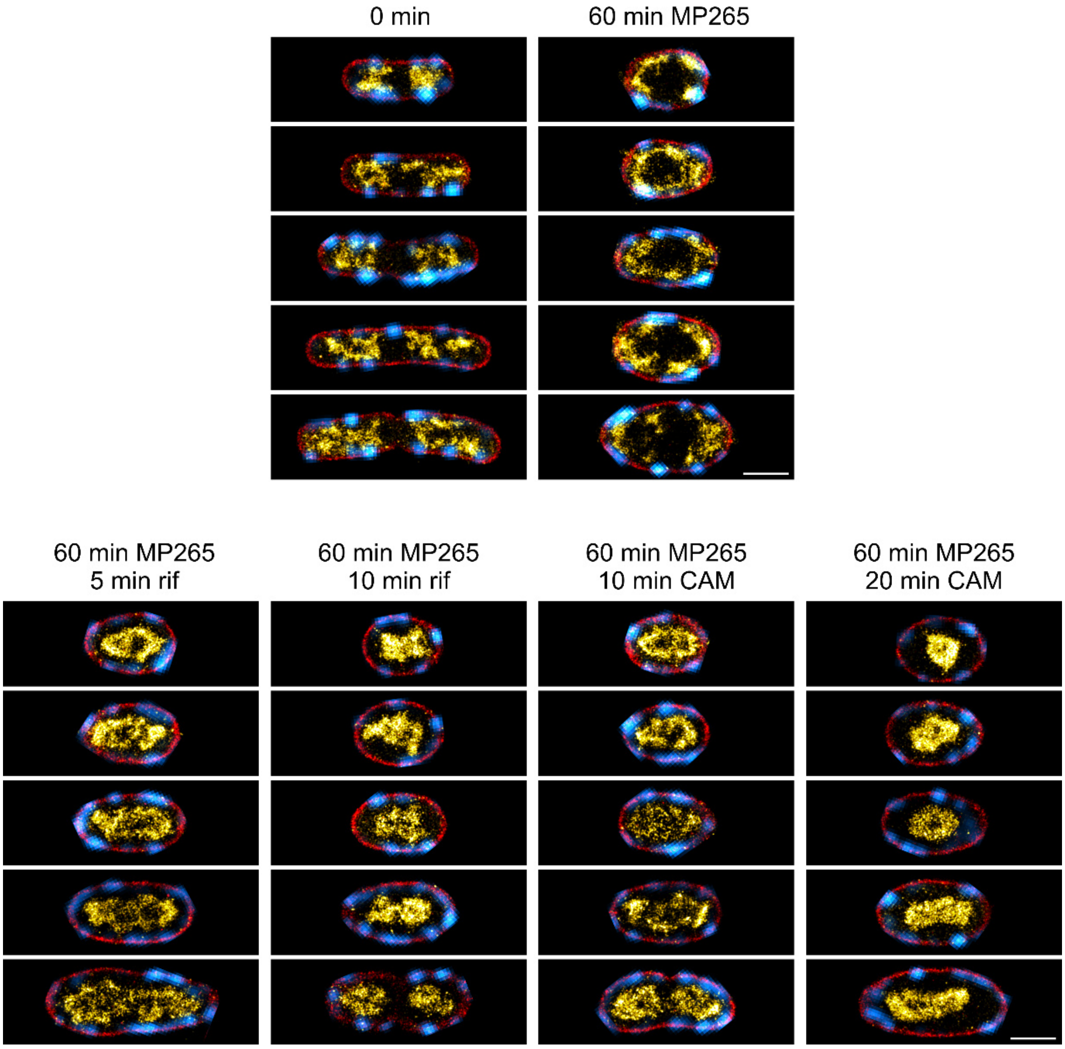

Figure S 28: Exemplary dual-color PAINT images of fixed E. coli NO34 cells treated with 25 µM MP265 and 100 µg/ml rifampicin or 50 µg/ml chloramphenicol. Scale bar is 1 µm.

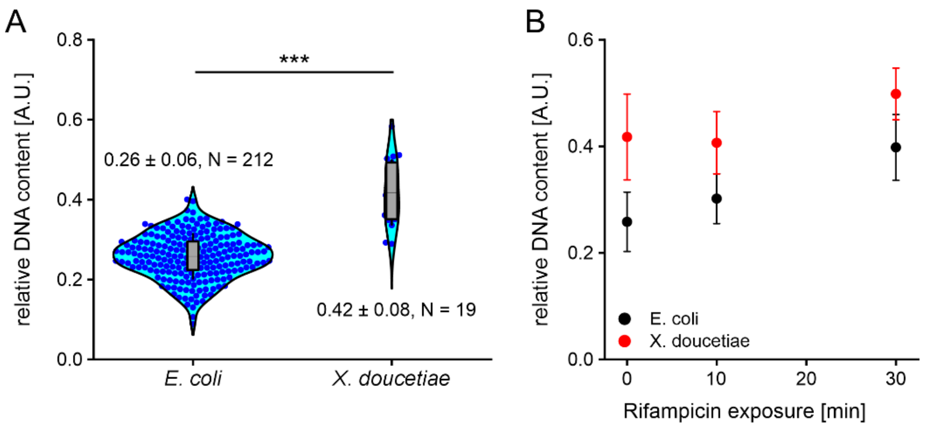

Figure S29: Comparison of the relative DNA content of exponentially growing X. doucetiae and E. coli cells. [A] Violin plot for untreated cells.Bars represent the 25%/75% percentiles. p = 2.95E-11 (Mann-Whitney-U test). [B] Relative DNA content in rifampicin treated cells. Source data are provided as a Source Data file.

Supplementary Tables

Table S1: Bacterial strains used in this study

Bacterial strain	Genotype	Reference/source
MG1655	*Escherichia coli* K-12 wild type	CGSG #6300
NO34	MG1655 *mreB^{sw}-sfGFP csrD::*kan	Gitai laboratory [3]
CS1	MG1655 *mreB^{sw}-sfGFP csrD::*kan *hupA::mScarlet-I-frt-cam-frt*	this work
CS2	MG1655 *mreB^{sw}-sfGFP csrD::*kan *hns::mScarlet-I-frt-cam-frt*	this work
CS_Xd1	*Xenorhabdus doucetiae* DSM17909 *mreB^{sw}-sfGFP*	this work
MBS106	NCM3416*, rne-frt*	Hadjeras *et al.* [4]
MBS157	NCM3416*, rneΔMTS-frt*	Hadjeras *et al.* [4]
SLP60	*Δrne-frt* + pKti4 (*lacIPO-rne-yfp*)	Kind gift from Agamemnon Carpousis

Table S2: Primers used in this study

Primer name	Sequence (5' – 3')
CS_FFM_002	CAACTGGTTGGTTTCGGTACC
CS_FFM_003	GAATTCACCAGAACCAGCAGCAGAACCAGCAGAACCCTTAACTGCGTCTTTCAGTGCC
CS_FFM_005	TGCTGCTGGTTCTGGTGAATTCGTGAGCAAGGGCGAGGC
CS_FFM_006	TTACTTGTACAGCTCGTCCATGCC
CS_FFM_012	AAGCTAAACGTGCTCAGCGTC
CS_FFM_013	GAATTCACCAGAACCAGCAGCAGAACCAGCAGAACCTTGCTTGATCAGGAAATCGTCG
CS_FFM_017	GGCATGGACGAGCTGTACAAGTAAGCATGGATGAGCTGTACAAATAAG
CS_FFM_018	CAGAAAGACAAAAGGGGTGAAACCACCCCTTCGTTAAAACTGTTCACTGCCACGCAAT CATTGTAGGCTGGAGCTGCTTC
CS_FFM_019	CAGAAAGACAAAAGGGGTGAAAC
CS_FFM_020	CAATAAAAAATCCCGCCGCTGGCGGGATTTTAAGCAAGTGCAATCTACAAAAGAATTG TAGGCTGGAGCTGCTTC
CS_FFM_021	CAATAAAAAATCCCGCCGCTGG
CS_MPI_003	ATCGATCCTCTAGAGTCGACCTGCACATCACCACAATTTTTCATCAC
CS_MPI_004	CGTGGGATAGGCAGAAC
CS_MPI_005	GATGAAGTACGGGAAATTGAAG
CS_MPI_006	TGGAATTCCCGGGAGAGCTCAGATCTTAACGAATTGAAAATTGACGACG
CS_MPI_018	TGAAATTGGTTCTGCCTATCCCACGTCTGGCTCGAGCAGTAAAGG
CS_MPI_019	GCACTTCAATTTCCCGTACTTCATC
CS_MPI_020	GAAGCTATCATCAATTATGTTCGC
CS_MPI_021	TGCTTCAAGAATTTCATTGGAATTCAG
VpDS132 fw	GATCGATCCTCTAGAGTCGACC
VpDS132 rv	CACATGTGGAATTGTGAGCGG

*Table S3: Count and length distribution of CLSM datasets used for cell averaging and determination of relative nucleoid lengths. Different columns indicate values for the individual replicates. Replicate 2 is shown in blue for clarity. * indicates release from Nalidixate treatment*

Drug	Time [min]	N		l_{mean} [µm]		s.d. [µm]		l_{min} [µm]		l_{max} [µm]	
Control	0	213	-	4.91	-	1.06	-	2.93	-	8.20	-
	2	161	-	4.98	-	1.20	-	3.19	-	9.32	-
	5	154	-	4.82	-	1.08	-	2.54	-	8.41	-
	10	165	-	4.84	-	1.13	-	3.16	-	10.10	-
	20	121	-	4.85	-	0.98	-	3.19	-	7.49	-
	30	146	-	4.56	-	0.96	-	2.89	-	7.46	-
	60	153	-	4.46	-	0.94	-	3.01	-	7.01	-
Azide	0	177	-	4.87	-	0.83	-	3.34	-	7.24	-
	2	136	-	4.87	-	0.83	-	3.15	-	7.63	-
	5	145	-	4.58	-	0.83	-	2.95	-	7.17	-
	10	200	-	4.39	-	0.88	-	2.95	-	7.24	-
	20	184	-	4.31	-	0.91	-	2.77	-	7.08	-
	30	194	-	4.19	-	0.88	-	2.75	-	6.75	-
	60	225	-	4.25	-	0.87	-	2.76	-	7.05	-
Rifampicin	0	187	274	5.17	4.88	1.04	0.98	3.60	2.85	9.51	9.25
	2	207	274	5.10	4.82	0.91	0.94	3.13	3.08	8.11	9.63
	5	179	239	4.91	4.64	0.96	0.91	3.20	2.96	7.13	7.44
	10	184	280	4.73	4.47	0.83	0.94	3.16	2.03	6.93	10.30
	20	246	303	4.44	4.13	0.93	0.85	2.75	2.80	7.08	8.60
	30	208	298	4.04	4.00	0.87	0.81	2.74	2.63	6.35	8.72
	60	364	349	3.80	3.77	0.78	0.67	2.22	2.60	5.97	6.50
CAM	0	219	315	5.15	4.05	1.11	0.79	3.34	2.78	10.50	6.21
	2	219	318	4.76	3.84	1.06	0.83	3.09	2.57	8.83	7.49
	5	212	202	4.68	3.86	1.03	0.73	2.96	2.65	8.65	6.65
	10	147	346	4.66	3.87	1.11	0.85	3.04	2.30	9.61	6.46
	20	163	186	4.40	3.71	1.08	0.73	2.67	2.60	7.84	6.38
	30	128	272	3.96	3.77	0.96	0.86	2.56	2.15	8.19	7.51
	60	193	234	3.77	3.66	0.80	0.60	2.58	2.38	6.35	5.59
MP265	0	183	185	5.05	4.87	0.89	0.95	3.17	2.99	7.54	7.51
	2	125	171	4.83	5.08	0.91	1.10	3.02	3.30	7.47	8.87
	5	129	140	4.90	4.86	0.87	1.03	3.35	3.33	7.79	7.76
	10	135	189	4.79	4.67	0.96	1.00	3.12	2.94	8.44	8.79
	20	178	95	4.36	4.58	0.90	1.10	3.03	3.01	7.59	9.62
	30	172	165	3.87	3.92	0.66	0.80	2.74	2.50	6.16	6.32
	60	231	210	3.01	3.20	0.53	0.67	2.19	2.23	5.22	6.28
Mecillinam	0	148	193	4.83	5.21	0.96	1.09	2.45	3.06	7.83	8.80
	2	90	214	4.89	5.17	1.12	1.07	3.32	3.40	7.73	8.68
	5	102	223	4.59	5.09	0.84	1.01	3.15	3.21	6.97	8.27
	10	125	155	4.59	4.74	0.88	0.93	3.17	2.99	7.71	7.63
	20	149	140	4.45	4.35	0.94	0.93	2.64	2.69	6.95	6.79
	30	147	164	4.04	3.90	0.79	0.74	2.75	2.82	6.63	7.31
	60	270	167	3.16	3.32	0.64	0.62	2.19	2.32	5.49	5.59
Nalidixate	0	182	171	5.23	5.34	0.96	1.10	3.56	3.38	7.83	8.65
	2	121	180	5.17	5.10	0.85	1.01	3.31	3.34	7.61	7.33
	5	154	172	4.96	5.04	0.90	1.14	3.18	3.20	7.89	8.21
	10	105	204	4.81	4.86	0.84	1.00	3.15	3.18	6.72	8.42
	20	137	170	5.03	4.75	1.25	0.92	2.94	3.17	9.11	8.82
	30	98	126	5.48	5.44	1.19	1.13	3.77	3.57	9.04	9.89
	60	90	-	8.25	-	2.31	-	4.96	-	14.10	-
	35 + 10*	-	76	-	6.37	-	1.36	-	4.34	-	10.50
	35 + 30*	-	34	-	10.40	-	2.24	-	6.05	-	14.90

*Table S4: Number of super-resolved E. coli cells used for erosion analysis. * indicates the nalidixate washout experiment*

Time [min]	MP265	Mecillinam	Azide	Rifampicin	CAM	Nalidixate
**0**	31	23	20	33	37	26
**2**	32	29	-	33	33	42
**5**	27	28	-	45	41	34
**10**	38	36	31	58	21	44
**20**	43	44	-	58	20	38
**30**	43	36	35	46	27	29
**60**	60	72	36	60	29	
**35 + 10 ***						21
**35 + 30 ***						14

Table S5: Number of super-resolved E. coli cells used for erosion analysis in multi-drug treatments

Time [min]	MP265	MP265 + Rifampicin	MP265 + CAM
**0**	39	-	-
**60**	66	-	-
**60 + 5**	-	57	-
**60 + 10**	-	53	57
**60 + 20**	-	-	52

Table S6: Macros and notebooks used in this study

Macro	Purpose
M1	Average PSF determination from bead stacks
M2	Smoothing of cell outlines in NR PAINT images
M3	Rotation, alignment and cell straightening – CLSM images
M4	Cell normalization – CLSM images
M5	Determination of the relative nucleoid length expansion – CLSM images
M6	Determination of the relative MreB distribution – CLSM images
M7	Determination of radial intensity distributions using erosion analysis
M8	Plot intensity profiles along perimeter
M9	Simulate images for circular cross-correlation
Notebook_1	Google Colab notebook for the calculation of circular cross-correlation

Table S7: Identifier of datasets made publicly available via Zenodo

Dataset	DOI
CLSM images of all treatments - *Escherichia coli*	https://doi.org/10.5281/zenodo.8430052
Data of length- and cross-axis plots (Figs. S5-8, Fig. S12)	https://doi.org/10.5281/zenodo.14967865
Nucleoid segmentation SMLM data	https://doi.org/10.5281/zenodo.8429932
SMLM images of all treatments – *Escherichia coli*	https://doi.org/10.5281/zenodo.8430032
SMLM images of *Xenorhabdus doucetiae*	https://doi.org/10.5281/zenodo.10007398
SMLM images of RNase E experiments	https://doi.org/10.5281/zenodo.14962042
Github Repository (v1.0)	https://doi.org/10.5281/zenodo.14968246

Supplementary References

1 Hardo, G., Li, R. & Bakshi, S. Quantitative microbiology with widefield microscopy: navigating optical artefacts for accurate interpretations. *Npj Imaging* **2**, 26, doi:10.1038/s44303-024-00024-4 (2024).

2 Palayret, M. *et al.* Virtual-'light-sheet' single-molecule localisation microscopy enables quantitative optical sectioning for super-resolution imaging. *PLoS One* **10**, e0125438, doi:10.1371/journal.pone.0125438 (2015).

3 Ouzounov, N. *et al.* MreB Orientation Correlates with Cell Diameter in Escherichia coli. *Biophys J* **111**, 1035-1043, doi:10.1016/j.bpj.2016.07.017 (2016).

4 Hadjeras, L. *et al.* Detachment of the RNA degradosome from the inner membrane of Escherichia coli results in a global slowdown of mRNA degradation, proteolysis of RNase E and increased turnover of ribosome-free transcripts. *Mol Microbiol* **111**, 1715-1731, doi:10.1111/mmi.14248 (2019).
