# Supplementary material for: The nucleoid of rapidly growing Escherichia coli localizes close to the inner membrane and is organized by transcription, translation, and cell geometry
Source: Nat Commun. 2025 Apr 20;16:3732. doi: 10.1038/s41467-025-58723-4 (PMC12009437; doi:10.1038/s41467-025-58723-4)
Supplement: Supplementary file 2 — Description of additional supplementary files [file 41467_2025_58723_MOESM2_ESM.pdf]

Description of Additional Supplementary Files:

Supplementary Movie 1: Rendering or 3D SMLM imaging of an E. coli cell click-labelled for DNA.

Supplementary Movie 2: Live-cell confocal microscopy time series of *E. coli* cells labeled for HU-α (HupA-mScarlet-I, red) and MreB (MreBsw-sfGFP, cyan). Scale bar is 2 μm.

Supplementary Movie 3: Live-cell confocal microscopy time series of *E. coli* cells labeled for H-NS (H-NS-mScarlet-I, red) and MreB (MreBsw-sfGFP, cyan). Scale bar is 2 μm.

Supplementary Movie 4: Live-cell confocal microscopy time series of *E. coli* cells labeled for H-NS (H-NS-mScarlet-I, red) and MreB (MreBsw-sfGFP, cyan). Cells were imaged under agarose pads containing 25 μM MP265. Time stamps on the left represent the starting point of the measurement after immobilization under the agarose pads, while stamps on the right indicate the time during the time lapse measurement. Scale bar is 2 μm.

Supplementary Software: Scripts and Macros used in this work. The Supplementary Software contains custom written Fiji macros M1-M9, as well as custom Python code used in this study.
