# Supplementary material for: The nucleoid of rapidly growing Escherichia coli localizes close to the inner membrane and is organized by transcription, translation, and cell geometry
Source: Nat Commun. 2025 Apr 20;16:3732. doi: 10.1038/s41467-025-58723-4 (PMC12009437; doi:10.1038/s41467-025-58723-4)
Supplement: Supplementary file 8 — Reporting summary [file 41467_2025_58723_MOESM8_ESM.pdf]

nature portfolio

Corresponding author(s): Christoph Spahn
Mike Heilemann

Last updated by author(s): Mar 5, 2025

Reporting Summary

Nature Portfolio wishes to improve the reproducibility of the work that we publish. This form provides structure for consistency and transparency in reporting. For further information on Nature Portfolio policies, see our Editorial Policies and the Editorial Policy Checklist.

Statistics

For all statistical analyses, confirm that the following items are present in the figure legend, table legend, main text, or Methods section.

n/a	Confirmed	
☐	☒	The exact sample size (*n*) for each experimental group/condition, given as a discrete number and unit of measurement
☐	☒	A statement on whether measurements were taken from distinct samples or whether the same sample was measured repeatedly
☐	☒	The statistical test(s) used AND whether they are one- or two-sided<br>*Only common tests should be described solely by name; describe more complex techniques in the Methods section.*
☒	☐	A description of all covariates tested
☒	☐	A description of any assumptions or corrections, such as tests of normality and adjustment for multiple comparisons
☐	☒	A full description of the statistical parameters including central tendency (e.g. means) or other basic estimates (e.g. regression coefficient) AND variation (e.g. standard deviation) or associated estimates of uncertainty (e.g. confidence intervals)
☐	☒	For null hypothesis testing, the test statistic (e.g. *F*, *t*, *r*) with confidence intervals, effect sizes, degrees of freedom and *P* value noted<br>*Give P values as exact values whenever suitable.*
☒	☐	For Bayesian analysis, information on the choice of priors and Markov chain Monte Carlo settings
☒	☐	For hierarchical and complex designs, identification of the appropriate level for tests and full reporting of outcomes
☒	☐	Estimates of effect sizes (e.g. Cohen's *d*, Pearson's *r*), indicating how they were calculated

Our web collection on statistics for biologists contains articles on many of the points above.

Software and code

Policy information about availability of computer code

Data collection	Images were acquired using the following systems and softwares:<br>Zeiss ELYRA PS1 (ZEN v14.0.20.201)<br>µManager v1.4.14;<br>Nikon NIS Elements ()<br>Zeiss LSM 710 (ZEN Blue)<br>Leica SP8 (LASX 3.5.5.19976)
Data analysis	SMLM data: rapidSTORM v3.31 and Picasso v.0.6.0<br>Fiji is just ImageJ (FIJI) (different versions, >1.4, latest 1.54f)<br>Spreadsheet analysis: OriginPro 9.1G (OriginLabs); Excel (Microsoft Office 365, Version 2409 Build 16.0.18025.20214)<br>Python 3<br>SuperPlotsOfData (https://huygens.science.uva.nl/SuperPlotsOfData/) (time of use: 2025-02-13)<br>Github repository: https://github.com/CKSpahn/Bacterial_image_analysis (deposited on Zenodo under 10.5281/zenodo.14968247)

For manuscripts utilizing custom algorithms or software that are central to the research but not yet described in published literature, software must be made available to editors and reviewers. We strongly encourage code deposition in a community repository (e.g. GitHub). See the Nature Portfolio guidelines for submitting code & software for further information.

Data

Policy information about availability of data

All manuscripts must include a data availability statement. This statement should provide the following information, where applicable:

- Accession codes, unique identifiers, or web links for publicly available datasets
- A description of any restrictions on data availability
- For clinical datasets or third party data, please ensure that the statement adheres to our policy

Confocal imaging data and multicolor super-resolution images for all conditions are available via Zenodo.
The CLSM images generated in this study have been deposited in the Zenodo database under accession 8430052 [https://zenodo.org/records/8430052].
The data of length- and cross-axis plots have been deposited in the Zenodo database under accession code 14967865 [https://zenodo.org/records/14967865].
SMLM image data of E. coli have been deposited in the Zenodo database under accession code 8430032 [https://zenodo.org/records/8430032].
SMLM image data of X. doucetiae have been deposited in the Zenodo database under accession code 10007398 [https://zenodo.org/records/10007398].
SMLM image data of E. coli RNase E experiments have been deposited in the Zenodo database under accession code 14962042 [https://zenodo.org/records/14962042].
SMLM nucleoid segmentation data and model have been deposited in the Zenodo database under accession code 8429932 [https://zenodo.org/records/8429932].
Raw single-molecule localization data will be shared upon request due to its extensive size. Source data are provided with this paper.

Research involving human participants, their data, or biological material

Policy information about studies with human participants or human data. See also policy information about sex, gender (identity/presentation), and sexual orientation and race, ethnicity and racism.

Reporting on sex and gender	*Use the terms sex (biological attribute) and gender (shaped by social and cultural circumstances) carefully in order to avoid confusing both terms. Indicate if findings apply to only one sex or gender; describe whether sex and gender were considered in study design; whether sex and/or gender was determined based on self-reporting or assigned and methods used. Provide in the source data disaggregated sex and gender data, where this information has been collected, and if consent has been obtained for sharing of individual-level data; provide overall numbers in this Reporting Summary. Please state if this information has not been collected. Report sex- and gender-based analyses where performed, justify reasons for lack of sex- and gender-based analysis.*
Reporting on race, ethnicity, or other socially relevant groupings	*Please specify the socially constructed or socially relevant categorization variable(s) used in your manuscript and explain why they were used. Please note that such variables should not be used as proxies for other socially constructed/relevant variables (for example, race or ethnicity should not be used as a proxy for socioeconomic status). Provide clear definitions of the relevant terms used, how they were provided (by the participants/respondents, the researchers, or third parties), and the method(s) used to classify people into the different categories (e.g. self-report, census or administrative data, social media data, etc.) Please provide details about how you controlled for confounding variables in your analyses.*
Population characteristics	*Describe the covariate-relevant population characteristics of the human research participants (e.g. age, genotypic information, past and current diagnosis and treatment categories). If you filled out the behavioural & social sciences study design questions and have nothing to add here, write "See above."*
Recruitment	*Describe how participants were recruited. Outline any potential self-selection bias or other biases that may be present and how these are likely to impact results.*
Ethics oversight	*Identify the organization(s) that approved the study protocol.*

Note that full information on the approval of the study protocol must also be provided in the manuscript.

Field-specific reporting

Please select the one below that is the best fit for your research. If you are not sure, read the appropriate sections before making your selection.

[x] Life sciences [ ] Behavioural & social sciences [ ] Ecological, evolutionary & environmental sciences

For a reference copy of the document with all sections, see nature.com/documents/nr-reporting-summary-flat.pdf

Life sciences study design

All studies must disclose on these points even when the disclosure is negative.

Sample size	Sample size was not determined a priori. We visually assessed the individual phenotypes of each experiment, revealing a highly reproducible phenotype between cells and experiments. The sample size was then chosed according to the resolution of the methods. For CLSM imaging, where differences were less obvious due to the low resolution, we chose a sample size of >50 cells (ideally 100 - 200 cells) per condition (~ 80 - 300). The absolute cell count varied due to differences in cell density on the glass surface. For SMLM experiments, we aimed for 20+ cells. (Exception: 35 min Release from nalidixate [14 cells], where the large cell length prevented recording of the full cell in a single field of view).

	Multiple images were recorded for each conditions and replicates to consider technical variation.
Data exclusions	Experiments were only conducted with bacterial cultures that showed unperturbed growth (mass doubling times of 25 - 30 min for E. coli). Data was only excluded when image quality was not meeting standards (large drift and shift between fluorescence channels). Individual cells were selected based on the following analysis criteria: Full attachment to the surface; no touching of other cells; correct z-focus position
Replication	CLSM experiments were conducted in duplicate, except for the non-treated control and azide treatment. Reproducibility of azide treatment was verified in independent test experiments using widefield imaging. Control culture showed reproducible results for the different time points, matching the untreated control in each drug treatment experiment. Multiple images were acquired for each condition. Super-resolution imaging of drug treated cells was performed on individual replicates that were selected from the CLSM replicates. Multiple images were recorded for each condition. RNase E experiments were performed in biological triplicates. Drug treaments reproducibly showed the expected phenotypes. Super-resolution imaging was only performed when cell morphology matched the phenotypes obtained by confocal imaging and the quality check using brightfield microscopy.
Randomization	During imaging, field of views were selected randomly while ensuring a sufficient number of individual cells per field of view.
Blinding	Bacteria were selected in brightfield imaging, thus preventing a bias generated by nucleoid or MreB structure/distribution.

Reporting for specific materials, systems and methods

We require information from authors about some types of materials, experimental systems and methods used in many studies. Here, indicate whether each material, system or method listed is relevant to your study. If you are not sure if a list item applies to your research, read the appropriate section before selecting a response.

Materials & experimental systems

n/a	Involved in the study	
☒	☐	Antibodies
☒	☐	Eukaryotic cell lines
☒	☐	Palaeontology and archaeology
☒	☐	Animals and other organisms
☒	☐	Clinical data
☒	☐	Dual use research of concern
☒	☐	Plants

Methods

n/a	Involved in the study	
☒	☐	ChIP-seq
☒	☐	Flow cytometry
☒	☐	MRI-based neuroimaging

Plants

Seed stocks	*Report on the source of all seed stocks or other plant material used. If applicable, state the seed stock centre and catalogue number. If plant specimens were collected from the field, describe the collection location, date and sampling procedures.*
Novel plant genotypes	*Describe the methods by which all novel plant genotypes were produced. This includes those generated by transgenic approaches, gene editing, chemical/radiation-based mutagenesis and hybridization. For transgenic lines, describe the transformation method, the number of independent lines analyzed and the generation upon which experiments were performed. For gene-edited lines, describe the editor used, the endogenous sequence targeted for editing, the targeting guide RNA sequence (if applicable) and how the editor was applied.*
Authentication	*Describe any authentication procedures for each seed stock used or novel genotype generated. Describe any experiments used to assess the effect of a mutation and, where applicable, how potential secondary effects (e.g. second site T-DNA insertions, mosiacism, off-target gene editing) were examined.*
